# Development and Prospects of Furin Inhibitors for Therapeutic Applications

**DOI:** 10.3390/ijms25179199

**Published:** 2024-08-24

**Authors:** Alexandre V. Ivachtchenko, Alexander V. Khvat, Dmitrii O. Shkil

**Affiliations:** ChemDiv Inc., San Diego, CA 92130, USA

**Keywords:** furin, PACE, PCSK3, PC1, PC3, PC1/3, proprotein convertase (PC), pan-protease inhibitor, serine protease inhibitor, viral proteins HIV, influenza, SARS-CoV-2, COVID-19, bacterial toxins, cancers

## Abstract

Furin, a serine protease enzyme located in the Golgi apparatus of animal cells, plays a crucial role in cleaving precursor proteins into their mature, active forms. It is ubiquitously expressed across various tissues, including the brain, lungs, gastrointestinal tract, liver, pancreas, and reproductive organs. Since its discovery in 1990, furin has been recognized as a significant therapeutic target, leading to the active development of furin inhibitors for potential use in antiviral, antibacterial, anticancer, and other therapeutic applications. This review provides a comprehensive overview of the progress in the development and characterization of furin inhibitors, encompassing peptides, linear and macrocyclic peptidomimetics, and non-peptide compounds, highlighting their potential in the treatment of both infectious and non-infectious diseases.

## 1. Introduction

**Figure 1:**
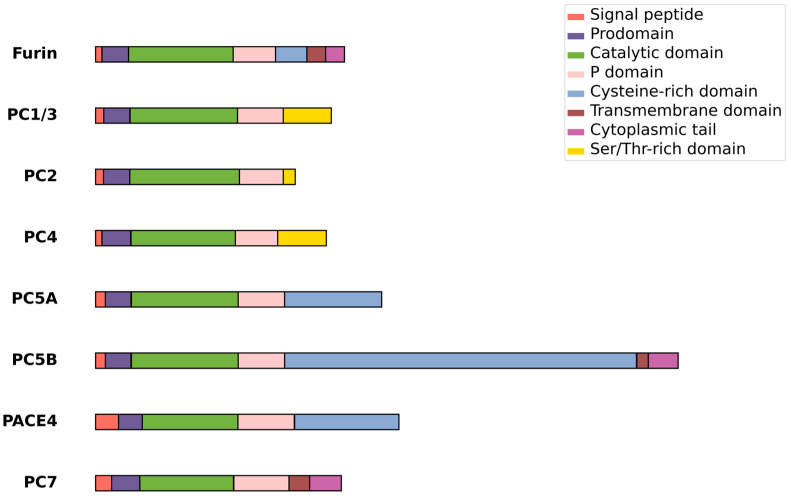
Kexin-like members of the PC family.

In recent years, the modulation of the activity of the cellular protease furin has emerged as a promising therapeutic approach for a variety of infectious and noninfectious diseases. Furin, previously known as the gene FUR (FES Upstream Region), is a serine protease ubiquitously expressed in all vertebrates and many invertebrates. It is also known as PACE (Paired basic Amino acid Cleaving Enzyme), PCSK3, and SPC1 and belongs to the proprotein convertase (PCs) family, which includes PC1 (also known as PC3 and commonly called PC1/3), PC2, PC4, PC5/PC6, PACE4, PC7, SKI-1/S1P, and PCSK9 [1,2,3]. Among these, PC1/3, PC2, PC4, PC5/6, PACE4, and PC7 are categorized as kexin-like proteases due to their structural and functional similarities to the yeast enzyme kexin (Figure 1). First identified in 1986 and later classified as an endoprotease, furin was the inaugural member of the proprotein convertase (PC) family to be recognized, with its distinct enzymatic identity confirmed in 1990 [4,5,6,7,8,9,10]. This calcium-dependent serine protease bears a structural resemblance to bacterial subtilisin and yeast kexin [11]. Furin is initially synthesized as an inactive proprotein, featuring an 83-amino-acid prodomain that is cleaved off, serving as an intramolecular chaperone to facilitate the enzyme’s activation and proper folding [12]. Predominantly located in the Golgi apparatus, furin plays a crucial role in the maturation of various precursor proteins, such as cell surface proteins, zymogens, hormones, and receptor proforms [13,14,15,16,17]. Its expression is widespread and found in all examined tissues and cell lines, including the brain, lungs, gastrointestinal tract, liver, pancreas, and reproductive tissues [17].

Furin cleavage sites are present in numerous proteins, including hormones, receptors, growth factors, and adhesion molecules. This protease is likely responsible for the cleavage and activation of over 150 substrates across mammals, viruses, and bacteria, including viral envelope glycoproteins, bacterial toxins, and cellular factors that, when hyperactivated, can promote tumor development and growth [18,19,20,21]. The minimal recognition sequence for furin cleavage is typically R-X-[K/R]-R↓. However, not every site that matches this sequence is cleaved by furin, and conversely, furin can sometimes recognize and cleave sites that do not perfectly adhere to this pattern. The furin cleavage recognition sequence extends over roughly 20 amino acids, ranging from position P14 to position P6′, and is comprised of two distinct segments: (1) a core region of eight amino acids (positions P6–P2′) that aligns with the furin-binding pocket, and (2) two polar regions—one spanning eight amino acids (positions P7–P14) and the other spanning four amino acids (positions P3′–P6′)—which are located outside the furin-binding pocket [6,8,22,23,24].

Furin processes many viral proteins necessary for viral entry and spread [25,26,27,28], including those from SARS-CoV-2 [29,30,31,32], avian influenza [33,34,35,36,37], HIV-1 [38,39,40,41,42,43,44], Chikungunya virus [45], HBV [46,47,48], HCV [49], influenza A, respiratory syncytial virus [50], human cytomegalovirus glycoprotein B (gpUL55) [51], Ebola virus [47,52,53], papilloma virus [54,55,56], and flaviviruses such as ZIKV and JEV [57]. In addition, host cell furin is involved in the activation of many bacterial toxins, including those from anthrax [58,59], diphtheria [59], Pseudomonas aeruginosa toxins [19,58,60], Shiga toxins [60], and dermonecrotic toxins from Bordetella species [61].

Overexpression of human furin is correlated with increased carcinogenic potential, contributing to the invasion and proliferation of cancers in the head and neck, breast, lungs, and other tissues [10,17,62,63,64,65,66,67,68,69]. Beyond cancer, furin activity is linked to the development of numerous human pathological conditions, such as cardiovascular diseases [70,71], rheumatoid diseases [72,73,74], and atherosclerosis [75,76].

Due to the ability of lethal viral and bacterial pathogens to hijack the furin pathway to enhance their virulence, there is a pressing need for therapeutic strategies that target furin. However, developing such strategies must carefully consider the widespread presence of furin throughout the human body, which raises concerns about potential toxicity from furin-inhibiting drugs [10]. Overexpression of furin has been linked to poorer outcomes in various cancers by promoting metastasis and reducing immune cell infiltration [10,77,78,79]. Thus, a deeper understanding of furin’s diverse functions in cancer could provide critical insights for developing effective cancer therapies targeting furin [68]. Moreover, bioinformatics studies have revealed that furin gene polymorphisms are associated with increased risks of diabetes, cardiovascular disease, obesity, and overall mortality [80,81]. These furin-related risk factors, combined with furin’s significant role in SARS-CoV-2 pathogenesis, may contribute to the greater vulnerability of certain populations—such as those with obesity or diabetes—to severe outcomes from COVID-19 [82,83]. Furin has been dubbed the “master switch of tumor growth and progression” [19,67,84] because its abnormal expression or activation can drive the development and progression of various cancers, including colon carcinoma, rhabdomyosarcoma, cancers of the head and neck, as well as tumors in the lung, skin, and brain. In some instances, furin levels have been found to correlate with increased tumor aggressiveness [77].

In 2022, Zhang et al. published a comprehensive review that explored the physiological functions of furin in the brain, emphasizing how the dysregulated expression of furin and its substrates is linked to neurodegenerative and neuropsychiatric disorders, including Alzheimer’s disease, Parkinson’s disease, epilepsy, cerebral ischemia, schizophrenia, and depression. The review also delves into the therapeutic implications of these findings and examines current strategies aimed at targeting furin for treatment purposes [85]. However, compared with infectious diseases [86,87,88,89,90,91] and cancer, the aberrant expression of furin and its pharmaceutical potential in neurological diseases remain poorly understood [85].

Nevertheless, even though furin inhibitors are considered an important class of biologically active compounds, we could not find any information on ongoing or completed clinical trials involving furin inhibitors. No furin inhibitors clinical trials were registered on the clinicaltrials.gov website or published in the Cortellis Drug Discovery Intelligence database (https://www.cortellis.com/drugdiscovery, accessed on 3 March 2024).

A noteworthy development is the series of phase I clinical trials investigating the safety and efficacy of a bi-shRNAi(furin)/GM-CSF DNA/autologous tumor cell vaccine (known as Vigil, FANG, or FANGTM), which has been genetically engineered to express GM-CSF and inhibit furin production [92,93,94,95,96,97,98,99]. The FANG expression vector was created in 2010 by Maples et al. [92], building on their earlier TAG vaccine vector [93]. FANG simultaneously expresses GM-CSF and a proprietary bifunctional shRNA targeting furin. Preclinical studies have demonstrated that inhibiting furin protein expression consequently prevents the activation of TGFβ1 and TGFβ2. Across various studies, treatment with the FANG vaccine in cases of advanced cancers [94]—such as Ewing’s sarcoma [98], liver cancer [96], and relapsed ovarian cancer [100], has shown long-term safety and potential benefits for patients with diverse forms of advanced malignancies [95]. Specifically, significant knockdown rates were observed for TGFβ1 (88–100%, with a mean of 98%), TGFβ2 (84–100%, with a mean of 95%), and furin (74–98%, with a mean of 89%) [101]. Long-term follow-up revealed that patients treated with the FANG vaccine survived for periods of 319, 729, 784, 931+, and 1043+ days [96]. Given the demonstrated long-term safety and positive outcomes in these studies, the authors advocate for further phase 2 clinical trials to evaluate the efficacy of FANG vaccine therapy.

In this review, we summarize the published data on the development of furin inhibitors for the treatment of infectious and non-infectious diseases.

## 2. Furin Inhibitors

Modulating furin activity with specific inhibitors offers a promising therapeutic strategy for both infectious and non-infectious diseases. A variety of physiological and bioengineered proteins, such as α1-PDX and its mutant variants, along with peptide, peptidomimetic, and non-peptide compounds, have been identified as furin inhibitors [29,102,103,104]. While the number of non-peptide furin inhibitors remains limited, recent years have seen the emergence of promising drug candidates in this category [105,106]. The significant accumulation of peptide, peptidomimetic, and small molecule furin inhibitors in the literature underscores the growing interest in this area, prompting a comprehensive review of the progress made.

Given furin’s involvement in various diseases, the development of potent and selective furin inhibitors has garnered considerable attention [14,21,29,31,91,107,108,109,110]. Designing effective furin-targeted therapies is challenging due to the need for inhibitors that are both highly selective for furin over other enzymes and specifically active in diseased tissues. The application of cheminformatics approaches, such as molecular modeling and quantitative structure–activity relationship (QSAR) models, which have demonstrated success in other areas of drug design [111,112,113,114,115], is now being extended to the study of furin inhibitors [89,116,117]. These methods facilitate the analysis of binding modes and the biological activity of potential inhibitors against the furin protein. The success of molecular modeling and machine learning techniques in the development of protease inhibitors and other therapeutic targets offers promising potential for significant advancements in the creation of effective furin inhibitors, thereby opening new avenues for the treatment of diseases associated with the dysfunction of this enzyme.

Currently, furin inhibitors are categorized as covalent or non-covalent and include peptides, peptidomimetics, and non-peptide inhibitors.

### 2.1. Peptidomimetic Furin Inhibitors

Peptides are widely used to target cancer cells, but their clinical utility is limited by issues such as low tissue penetration and immunogenicity. Peptidomimetics offers a promising alternative, with recent advances in synthetic production and computational biology enhancing their effectiveness in cancer and viral disease research [106].

#### 2.1.1. Covalent Peptidomimetic Furin Inhibitors

Peptidomimetic covalent inhibitors, which are peptidyl chloromethyl ketones (CMK), first appeared in the early 1960s [118]. In that and subsequent works [119,120,121,122,123], the inhibitory properties of the peptidyl-CMKs interacting with the active centers of trypsin-like enzymes to form covalent bonds were studied. It was found that a number of tripeptides with a C-terminal Lys-CMK motif inhibit the activity of subtilisin, thrombin, and plasma kallikrein. In particular, Ala-Phe-Lys-CMK proved to be an enzyme-specific inhibitor against individual representatives of the trypsin-like group. It can be easily inactivated by plasma kallikrein but not thrombin [120,121].

Significant specificity for the inactivation of “trypsin-like” enzymes involved in coagulation and fibrinolysis was obtained by modifying the peptidyl fragment of the reagents [122,124]. Ala-Phe-Arg-CMK and Pro-Phe-Arg-CMK were found to be highly potent and selective inhibitors for human plasma kallikrein. Other trypsin-like proteases were less sensitive to these inhibitors; in particular, plasmin was 48 times less sensitive, and factor Xa, thrombin, and urokinase were 102 to 105 times less sensitive. The affinity of human plasma kallikrein for Ala-Phe-Arg-CMK (K_i_ = 0.078 µM) is approximately 60 times higher than for Ala-Phe-Lys-CMK (K_i_ = 4.9 µM), while human plasmin exhibits approximately the same affinity for the first affinity label (K_i_ = 1.3 µM), as well as to the second (K_i_ = 0.83 µM) [124]. It was also shown that DNS-Glu-Gly-Arg-CMK and Ac-Glu-Gly-Arg-CMK were the most selective inhibitors of factor Xa, being 16–22 times more effective than human plasma kallikrein and at least 50 times more effective than thrombin and plasmin [123].

The spike glycoproteins of many enveloped viruses, including pathogenic avian influenza viruses, human parainfluenza virus, human cytomegalovirus, and human immunodeficiency virus, are proteolytically cleaved at the carboxyterminal of sequences containing the basic motif R-X-K/R-R and are activated by furin. The first generation of furin inhibitors—peptidoyl-chloromethyl ketones (CMK)—including Ala-Lys-Arg-CMK (AKR-CMK), Tyr-Ala-Lys-Arg-CMK (YAKR-CMK), Phe-Ala-Lys-Arg-CMK (FAKR-CMK), Ala-Phe-Arg-CMK (AFR-CMK), palmitoyl-FAKR-CMK (pal-FAKR-CMK), and pal-FAKR-chloroethyl ketone (pal-FAKR-CEK)—appeared in 1989 [125], almost simultaneously with the discovery of furin.

The inhibition by peptidoylchloroalkylketones of the activation of hemagglutinins of the fowl plague virus by proteases specific to paired basic residues was studied, and it was found that an increase in the inhibitory activity by 100–200 times in intact cells can be achieved due to the N-terminal acylation of peptidoylchloroalkylketones [125].

Later, Stieneke-Gröber et al. [126] on other objects confirmed the discovery of Garten et al. [125] about the higher inhibitory activity of polypeptididoyl-CMK containing an acyl fragment compared to unacylated polypeptididoyl-CMC. Among the studied inhibitors (AKR-CMK, YAKR-CMK, pal-FAKR-CEK, dec-REKR-CMK, dec-RAIR-CMK, and dec-FAKR-CMK) was found to be the most active against the HE influenza virus A/FPV/Dutch/December 27 (H7N7) RECR-CMK.

**Figure 2:**
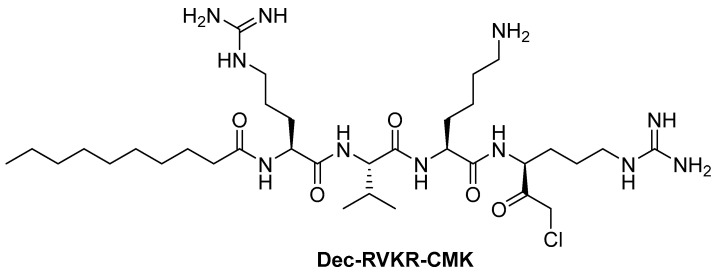
Covalent Dec-RVKR-CMK furin inhibitor.

Among the numerous polypeptidoyl-CMK inhibitors of furin [127], Dec-RVKR-CMK (Figure 2) emerged as the most attractive [41,57,105], the synthesis of which and its effect on the yeast Kex2 proteinase were first published in 1993 [128].

Dec-RVKR-CMK is a highly specific, potent, covalent and cell-permeable competitive inhibitor of proprotein convertases with K_i_ values of ~1 nM against furin/SPC1, 0.36 nM against SPC2/PC2, 2.0 nM against SPC3/PC1/PC3, 3.6 nM against SPC4/PACE4, and 0.12 nM against SPC6/PC5/PC6 and SPC7/LPC/PC7/PC8 [129]. According to Douglas et al., Dec-RVKR-CMK has IC_50_ values of 1.3 ± 3.6 nM against furin, 0.17 ± 0.21 nM against proprotein convertases subtilisin/kexin-type PCSK5, 0.65 ± 0.43 nM against PCSK6, 0.54 ± 0.68 nM against PCSK7, and in vitro Golgi inhibitory activity determined in U2OS cells is 9108 ± 6187 nM [105]. It also prevents the cleavage of the SARS-CoV-2 spike protein by furin, thereby blocking viral entry into cells (IC_50_ = 57 nM in a plaque reduction assay) [86].

Dec-RVKR-CMK acts as an antiviral agent against various viruses [38,45,47,49,50,51,54,57]; however, CMKs are not suitable for drug development because they are prone to racemization at the Cα carbon, including reactive chlorine, and therefore can be easily attacked by numerous nucleophiles, limiting their selectivity and stability in vivo [127].

**Figure 3:**
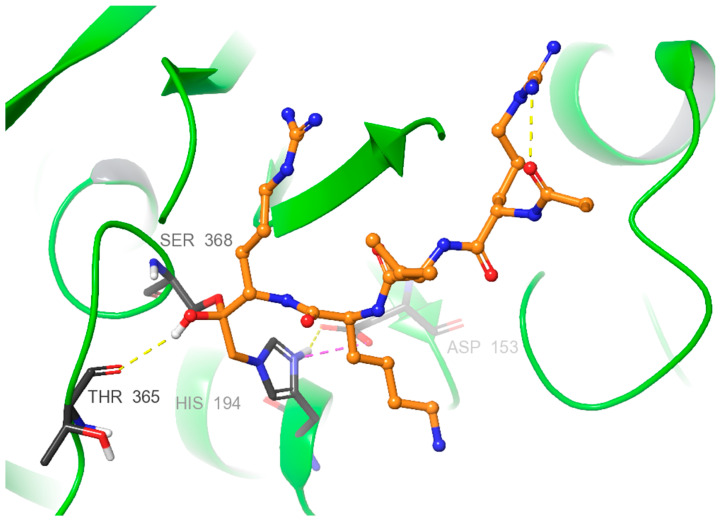
Mechanism of interaction between Dec-RVKR-CMK and furin (PDB ID: 5JMO) is illustrated, with key amino acid residues represented by black carbon sticks and the Dec-RVKR-CMK carbons depicted in orange. Hydrogen bonds are shown as yellow dotted lines, while salt bridges are indicated by purple dotted lines. Green helices represent the furin protein.

X-ray diffraction studies of the complexes furin:Dec-RVKR-CMK [130] and furin:Dec-RVKR-CMK:Nb14 [131] allowed us to establish the mechanism of interaction between Dec-RVKR-CMK and furin. These investigations highlight that the key moieties Ser368, His194, and Asp153 form an active center triad crucial for the interaction with covalent inhibitors such as Dec-RVKR-CMK. This interaction is characterized by the binding of the Ser368 alcohol group to the carbonyl carbon of Dec-RVKR-CMK, forming a hemiketal tetrahedral intermediate. His194 is covalently linked by replacing the chlorine atom in Dec-RVKR-CMK with the nitrogen from His194’s backbone group. This complex stabilization is presumably facilitated by electrostatic compensations between the positive charge on His194’s imidazole nitrogen and Asp153’s carboxyl group, as well as the H-bond of hemiketal with Thr365 (Figure 3).

#### 2.1.2. Non-Covalent Polypeptide Furin Inhibitors

Some of the first non-covalent polypeptide inhibitors of PCs were pseudopeptides incorporating aminomethylene (CH_2_NH) [132], ketomethylene COCH_2_, or aminomethylketone COCH_2_NH moieties [133], which appear to inhibit furin through the formation of a reversible hemiketal. The L-peptidomimetic Dec-Arg-Val-Lys-L-Arg-CH_2_-Ala-Val-Gly-NH_2_ with K_i_ = 3.4 nM was found to be the most active in this series of inhibitors. The K_i_ of the latter is two orders of magnitude lower than that of the ketomethylene analogue Dec-Arg-Val-Lys-Arg-C(O)CH_2_-Gly-Val-Gly-Ile-OMe. It is noted that aminomethylketone-containing peptidomimetics are resistant to enzymatic degradation; this keto group can form a tetrahedral hemiketal with a serine hydroxyl at the active site, and the amino acid sequence on the carbonyl side of these bonds can increase the binding affinity of the inhibitor. The D-peptidomimetic Dec-Arg-Val-Lys-D-Arg-CH2-Ala-Val-Gly-NH_2_ was also found to inhibit furin, but with a K_i_ value that was 7.6 times higher than that of the L-isomer [133].

Later on, Apletalina et al. utilized a combinatorial position-scan synthetic peptide library comprising approximately 52 million hexapeptides to identify potential inhibitory peptides for recombinant mouse prohormone convertases PC1 and PC2, as well as to gather insights into the specificity of these enzymes. Using the information obtained from surveys of this library, we synthesized sets of hexapeptides and tested them for inhibition of PC1, PC2, and furin. The authors showed that both enzymes prefer a P3 Val. In this case, many substitutions at S5 were well tolerated, and the selectivity of PC1 and PC2 depended mainly on their S6 position. Among the studied series of hexapeptides, the most active inhibitor against PC1 was Ac-Leu-Leu-Arg-Val-Lys-Arg-NH_2_, with K_i_ = 3.2 ± 1.0 8 nM, while against PC2 and furin, it showed K_i_ values of 360 ± 50 and 1400 ± 230 nM, respectively. The maximum activity against furin was shown by Ac-Leu-Lys-Arg-Val-Lys-Arg-NH_2_ with K_i_ = 190 ± 20 nM, while against P1 and PC2 furin, it showed K_i_ values of 5.7 ± 1.5 and 620 ± 150 nM, respectively [134].

Plasma membranes play a critical role in maintaining cellular structures and functions. They serve as a strong barrier to the intracellular delivery of many agents. However, arginine-rich polypeptides have been shown to penetrate cells well and are, therefore, widely studied as vectors for the delivery of membrane-permeabilizing agents into cells [135,136,137,138,139,140,141,142,143,144,145,146,147,148,149,150].

Cameron et al. studied combinatorial libraries of amidated and acetylated L- and D-hexapeptides, which were found to be micromolar furin inhibitors with the K_i_ values for L-hexapeptides ranging from 1.3 ± 0.9 μM for Ac-RRKRRR-NH_2_ to 13.2 ± 1.6 μM for Ac-HHKRRR-NH_2_, and K_i_ for D-hexapeptides from 2.4 ± 0.8 μM for Ac-KRKRRR-NH_2_ to 22.7 ± 4.3 μM for Ac-WRRRIL-NH_2_. It was found that L-Arg or L-Lys at all positions of the hexapeptides are more active inhibitors of furin. For PC2, the K_i_ values for this series of amidated and acetylated L- and D-hexapeptides ranged from 152 ± 30 μM for Ac-RHKRRR-NH_2_ to 1500 ± 300 μM for Ac-MRKRRR-NH_2_ [151].

It was also found that terminal groups of hexapeptides reduce the effectiveness of inhibition; in particular, the substituted furin inhibitors LLRVKR-NH_2_, LLRVKR-NH_2_, and Ac-LLRVKR-NH_2_ had K_i_ values of 0.8 ± 0.1, 0.8 ± 0.1, and 3.4 ± 0.1 μM, respectively, and the corresponding unsubstituted hexapeptide LLRVKR had K_i_ = 0.42 ± 0.02 μM. In this regard, the authors obtained a series of L-polyarginines, including those with four to nine arginine moieties and hexa-D-arginine (Table 1). As the chain length increased from four to nine arginine moieties, the K_i_ values for furin inhibition of L-polyarginine increased from 42 nM for nona-L-arginine to 6.4 µM for tetra-L-arginine. While the K_i_ values for the nona-, octa-, hepta-, and hexamers varied between 42 and 114 nM, there was an approximately 10-fold increase in K_i_ between the hexamer and heptamer, and a 5-fold rise between the heptamer and tetramer.

D6R has been reported to reduce the toxicity of Pseudomonas aeruginosa exotoxin A (PEA) both in cell culture and in living animals without causing a cytokine response [152]. It also protects against anthrax toxicosis both in vivo and in vitro [153], reduces the secretion of hepatitis B e-antigen in patients with chronic hepatitis B viral infection, and helps reduce immune tolerance [47]. Additionally, D6R increases 7B2•PC2 activity in hyp-mouse osteoblasts and rescues the HYP phenotype [154], suppresses the proliferation and epithelial–mesenchymal transition (EMT) of pancreatic cancer cells, and acts as an ideal compound for the treatment of pancreatic cancer [155].

D6R is noted for its other potentially promising therapeutic properties, including its ability to cross cell membranes, its small size that allows it to achieve useful therapeutic concentrations, and its lack of apparent cytotoxicity [152]. The therapeutic use of D-polyarginines is especially interesting because they are not cleaved by furin and possess inhibitory potency almost equal to that of L-polyarginine [156].

**Figure 4:**
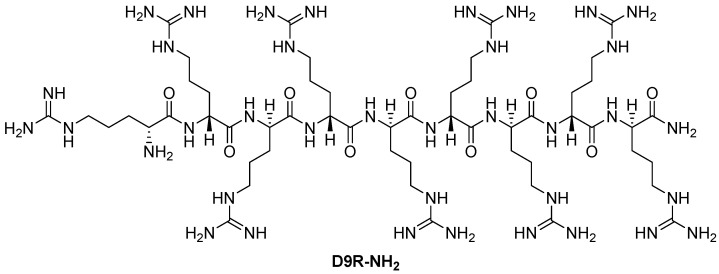
Furin inhibitor nona-D-arginine amid (D9R-NH_2_).

Kacprzak et al. [156] further studied the inhibitory properties of L- and D-polyarginines against human furin. Although L9R can be cleaved by furin, it remains a significantly more potent inhibitor than D6R amide and L6R [151]. The authors examined the inhibitory efficacy of D-polyarginines of varying lengths against human furin and found that D-peptides were not cleaved by furin. Consistent with previous findings using L-peptides [151], increasing chain length resulted in an increase in their furin inhibition efficiency, resulting in an extremely low K_i_ value of 1.3 ± 0.2 nM in the case of D9R amide (D9R-NH_2_, Figure 4). The authors also found that the transition from D6R to D6R-NH_2_ led to an eightfold increase in the efficiency of furin inhibition of the latter (K_i_ values of 142 and 24 nM, respectively) [156].

Becker et al. [157] synthesized and studied a series of furin-inhibiting peptidomimetics with the general formula P5-Arg-Val-P2-P1, containing decarboxylated arginine mimetics as the P1 moiety, which had been previously studied in other PC inhibitors [28,42,128,134,151,156,158,159], and used Arg and Lys as the P2 fragment (Table 2). As a result, the 4-amidinobenzylamide (4-Amba) moiety was identified as an excellent replacement for arginine. The most potent compound, phenylacetyl-Arg-Val-Arg-4-amidinobenzylamide (Phac-Arg-Val-Arg-4-Amba), inhibited furin with a K_i_-value of 0.81 nM (Table 2) and has comparable affinity to other PCs like PC1/3, PACE4, and PC5/6, for which the K_i_-value had a value of 0.75, 0.6, and 1.6 nM, respectively. At the same time, PC2, PC7, and trypsin-like serine proteases were less affected. K_i_ values of PC2, PC7, thrombin, fXa, and plasmin were 6.154, 0.312, 23, 40, and 6 μM, respectively (Table 3). In fowl plague virus (influenza A, H7N1)-infected MDCK cells, inhibitor 15 reduced proteolytic hemagglutinin cleavage and was able to reduce virus propagation in a long-term infection test.

Somewhat later, Becker et al. synthesized several new substrate analogue furin inhibitors with the 4-Amba moiety as P1, which have furin inhibition constants in the low nanomolar range (Table 4). Due to the close structural similarity of these synthesized compounds to the previously described inhibitor **15** [158], which is also a highly potent inhibitor of PC1/3, PC5/6, and PACE4, the authors hypothesize that at least some of these analogues should also inhibit other PCs.

Further optimization of the moiety P5 in inhibitor **15** led the authors to develop picomolar furin inhibitors (Table 5), the most active of which have a K_i_ value < 10 pM [28,159]. According to the authors, the most potent furin inhibitors, **24** (K_i_ = 16 pM) and **26** (K_i_ = 8 pM), were highlighted as potentially valuable tools for targeting the active sites of PC1/3, PC4, PACE4, or PC5/6 (Table 6). Their remarkable potency enables significant inhibition at concentrations lower than those of the enzyme in the assay. Overall, the analogues tested in this series displayed limited selectivity for other furin-like proprotein convertases (PCs), such as PC1/3, PC4, PACE4, or PC5/6. While high specificity for the target enzyme is generally preferred to minimize side effects, there are cases where inhibiting multiple PCs may be advantageous to prevent compensatory mechanisms by other PCs in pathological conditions. Additionally, the low affinity of these analogues for trypsin-like serine proteases (such as thrombin) is advantageous for preserving blood homeostasis. Subsequent studies examined the X-ray structures of human furin complexes with inhibitors **2** (Phac-RVR-GABA) [160], **24** (4-Huame-Phac-RVR-GAMBA) [28,161], and **26** (3-GUAME-PHAC- RVR-Amba) [160].

Inhibitor **24** was shown to significantly reduce the replication of the highly pathogenic avian influenza virus strain H7N1 compared to a control without the inhibitor [28]. It also notably enhanced the antiviral effects of oseltamivir and ribavirin against strains of highly pathogenic avian influenza viruses A/Thailand/1 (KAN-1)/2004 (H5N1) and A/FPV/Rostock/1934 (H7N1) in MDCK cells, as well as delayed the development of resistance to oseltamivir [35]. Additionally, it proved effective in protecting HEp-2 cells from intoxication by Shiga toxin [28].

**Figure 5:**
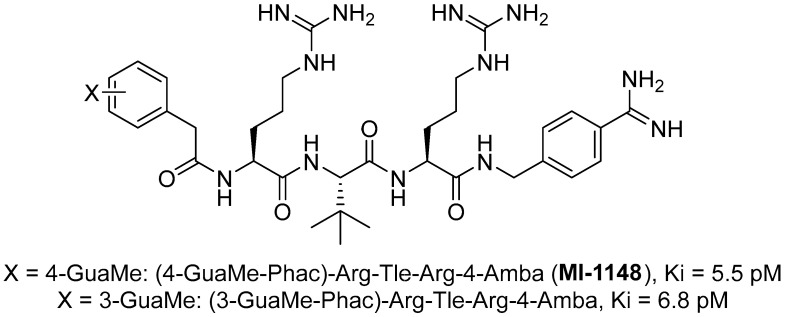
General formula of furin inhibitors from the group led by Prof. T. Steinmetzer [161,162,163,164].

The group led by Prof. T. Steinmetzer [161,162,163,164] optimized previously described peptidomimetic furin inhibitors [28,157,158] by changing P3, P5, and P1 moieties, resulting in new, highly effective picomolar furin inhibitors (Figure 5). These furin inhibitors also strongly inhibit PC1/3, while PC2 is less affected.

MI-1148 and its derivatives have been shown to inhibit the replication of various furin-dependent viruses in cell culture, including highly pathogenic avian influenza strains H5N1 and H7N1 [28,35,162], anthrax and diphtheria toxin [161], chikungunya virus [162], canine distemper virus [161], Dengue or West Nile virus [163,164], mumps virus [165], and respiratory syncytial virus [166]. MI-1148 and its analogues showed only minor toxicity in all cell cultures used, up to concentrations of 50 μM, but they showed significant toxicity in mice [163,167].

**Figure 6:**
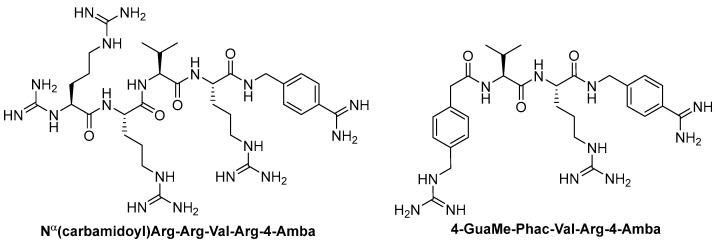
Furin inhibitors from the article [152].

Further optimization of the structure [162] of the known furin inhibitor Phac-Arg-Val-Arg-4-Amba [157] led to the development of both picomolar extended and nanomolar shortened inhibitors. The strongest in the studied series of inhibitors was Nα(carbamidoyl)Arg-Arg-Val-Arg-4-Amba with K_i_ = 6.2 pM, containing an additional main moiety at the N-terminus, and the weakest was 4-GuaMe-Phac-Val-Arg-4-Amba, with K_i_ = 269.3 nM (Figure 6). The research demonstrated that extending the P4-P1 Arg-Val-Arg-4-Amba segment with additional basic residues significantly enhances the effectiveness of furin inhibitors. Some analogues displayed considerable activity in cell culture assays, whereas those lacking the P5 residue—designed to reduce molecular weight—still inhibited furin in the nanomolar range but failed to exhibit antiviral activity. Additionally, some of these new inhibitors were effective in protecting cells from the cytotoxic effects of diphtheria toxin [162].

The binding mode of MI-1148 in complex with furin was characterized by crystal structure determination [161]. The 4-Amba moiety binds in the S1 pocket and participates in multiple contacts with furin, thereby contributing to the picomolar activity of the MI-1148 analogues. The 4-Amba moiety has also been used to develop N-terminally extended furin and PACE4 inhibitors [166,168]. MI-1148 did not exhibit significant cytotoxicity in cell culture tests, but significant toxicity was observed in mice [166]. In this regard, Ivanova et al. suggested that this toxicity was caused by the highly polybasic nature of the molecule and the presence of a moiety at the P14-Amba position, which they replaced with a less basic moiety. Some of the resulting furin inhibitors showed relatively potent inhibition of furin, but only weak inhibitory effects were observed in cells. The authors, as a second approach to reducing the basicity of the inhibitors, retained the P1 4-Amba moiety but replaced P2 arginine with lysine. The obtained furin inhibitor, 4-GuaMe-Phac-Arg-Tle-Lys-Amba, with K_i_ = 8.5 nM, showed slightly reduced efficiency compared to MI-1148 but exhibited similar antiviral activity against West Nile and Dengue viruses in cell culture and reduced toxicity in mice by half [163].

**Figure 7:**
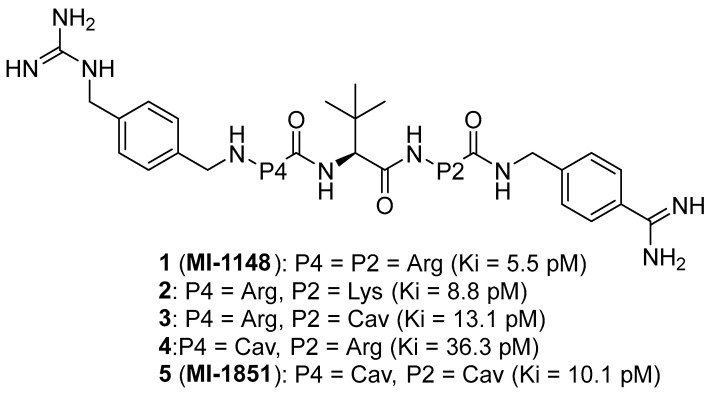
Canavanine-containing furin inhibitors.

Later, Lam van et al. [167] suggested that the toxicity of the compound MI-1148 is due not only to the P1 Amba moiety but also to the presence of three strongly basic guanidine groups and concluded that it was necessary to replace the strongly basic arginine P2 and P4 moieties in MI-1148 with less basic canavanine (Cav) fragments, containing a weakly basic oxyguanidine group with a pKa value of 7.01. Similar guanidine groups, as noted by the authors, had previously been included in some thrombin inhibitors to improve their bioavailability [169]. This strategy allowed the authors to obtain new furin inhibitors (Figure 7) that bind to furin in crystal structures similar to their arginine analogues. They have comparable antiviral activity in cell culture compared to MI-1148 but with markedly reduced toxicity in mice [167].

The canavanine-containing furin inhibitors studied have demonstrated significant antiviral activity against furin-dependent viruses such as RSV, WNV, and Dengue-2 virus. Among these, MI-1851 (Ki = 10.1 pM) showed the greatest efficacy against RSV and effectively inhibited the replication of West Nile virus and Dengue-2 virus while exhibiting significantly reduced toxicity in mice and rats compared to the inhibitor MI-1148.

MI-1851 has also been demonstrated to reduce SARS-CoV-2 replication in infected Calu-3 cells by inhibiting the S1/S2 cleavage of the spike protein [170]. Additionally, researchers found that combining TMPRSS2 inhibitors, such as aprotinin or MI-432, with the furin inhibitor MI-1851 enhanced antiviral efficacy against SARS-CoV-2 in human respiratory tract cells. This combination significantly lowered viral replication at reduced doses compared to the use of each inhibitor individually. As a result, the authors propose that the combined use of TMPRSS2 and furin inhibitors could be a promising therapeutic approach for treating SARS-CoV-2 infections, potentially enhancing antiviral effects while minimizing drug toxicity and side effects by allowing for lower doses of the inhibitors [170,171]. Furthermore, MI-1851 did not inhibit the enzymes CYP1A2, 2C9, 2C19, or 2D6. In human hepatocytes, MI-1851 significantly inhibited CYP3A4, with only weak inhibition observed against human microsomes and recombinant human CYP3A4. Importantly, MI-1851 did not affect the viability or oxidative status of primary human hepatocytes, even at concentrations as high as 100 μM. Based on these findings, the furin inhibitor MI-1851 emerges as a potential drug candidate for the treatment of COVID-19, given furin’s crucial role in the priming and activation of the SARS-CoV-2 spike protein [171].

**Figure 8:**
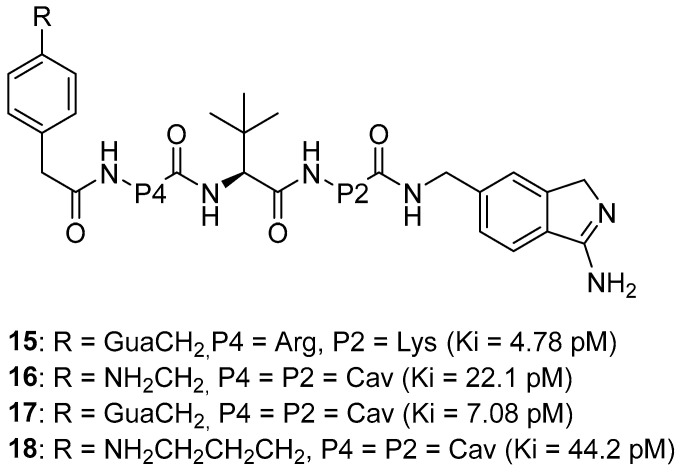
P1 3-amino-1H-isoindol-6-yl-containing furin inhibitors.

Recently, this group of researchers published further optimization of the furin inhibitors MI-1851 (K_i_ = 10.1 pM) and its analogues [167] by replacing the P1 4-Amba moiety with the P1 1H-isoindol-3-amin moiety in these inhibitors (Amia) (Figure 8). The P1 Amia was identified as an optimal P1 group for proprotein convertase furin through crystallographic screening of 20 fragments known to occupy the S1 pocket of trypsin-like serine proteases. The most active derivatives in this series of the P1 Amia were inhibitors 15 (K_i_ = 4.78 pM) and 17 (K_i_ = 7.08 pM). The crystal structures of these inhibitors in complex with furin, determined using X-ray crystallography, showed that the new P1 moiety is ideally suitable for inclusion in peptide furin inhibitors [172].

Inhibitor 15 effectively inhibited respiratory syncytial virus (RSV) strain A2, similar to inhibitor MI-1851. However, the greatest effectiveness was found in inhibitor 17, which reduced virus titers by more than 2000 times at a concentration of 1 μM and completely blocked virus replication at a concentration of 2.5 μM. Inhibitors 16 and 18 showed the least antiviral activity, nevertheless causing a decrease in virus titer by 1000 and 100 times, respectively.

Inhibitors 15, 17, and the reference inhibitor MI-1851 effectively inhibited the activity of highly pathogenic avian influenza A viruses SC35M in A549 cells, which replicate efficiently in mammalian cells. Inhibitor 15 demonstrated the strongest antiviral activity and reduced viral titers by 10,000-fold at 48 and 72 h. The efficacy of inhibitor **17** was between that of inhibitors 15 and MI-1851, while reduced efficacy was found for inhibitors 16 and 18.

The toxicity of inhibitor 17 in mice is comparable to that of the MI-1851 inhibitor, which is explained by the structural similarity between compounds MI-1851 and 17, with the exception of P1, and allowed the authors to suggest that the P1 fragment of Amia has the same effect on the toxicity profile of these inhibitors in mice as the P1 fragment of Amba [172].

Polypeptidomimetics represents a promising therapeutic class of drug candidates due to their high biological activity and specificity for biological targets. However, their main disadvantages are high conformational flexibility, susceptibility to proteolytic degradation leading to a short half-life (t1/2), and low bioavailability. A classic strategy to overcome some of these disadvantages is to modify them through macrocyclization [173,174,175,176,177]. Between 2001 and 2021, 18 cyclic peptides were approved for clinical use [176]. An average of around one cyclic peptide drug was approved per year [173].

#### 2.1.3. Non-Covalent Macrocyclic Peptidomimetic (MCPP) Furin Inhibitors

Macrocyclic Polypeptides (MCPPs) are polypeptide chains composed of both canonical and non-canonical amino acids connected at distant positions. These compounds have garnered significant interest from researchers due to their distinct characteristics. The three-dimensional structures of MCPPs provide a diverse array of unique shapes, enabling exceptional interactions with biological targets. The potential of MCPPs as therapeutic agents has sparked considerable excitement, driven by the success of developing potent drugs based on naturally occurring MCPPs. These naturally derived compounds are prevalent in nature and have been utilized as molecular frameworks for drug development. Today, more than 40 MCPPs of natural origin or their derivatives are used as therapeutic agents.

Compared with linear analogues, MCPPs have high affinity and specificity for binding, proteolytic stability, and, in some cases, improved penetration through the membrane. The beneficial properties of MCPPs and the development of drugs based on them are discussed in detail in a recently published review [173].

##### Natural MCPPs

**Figure 9:**
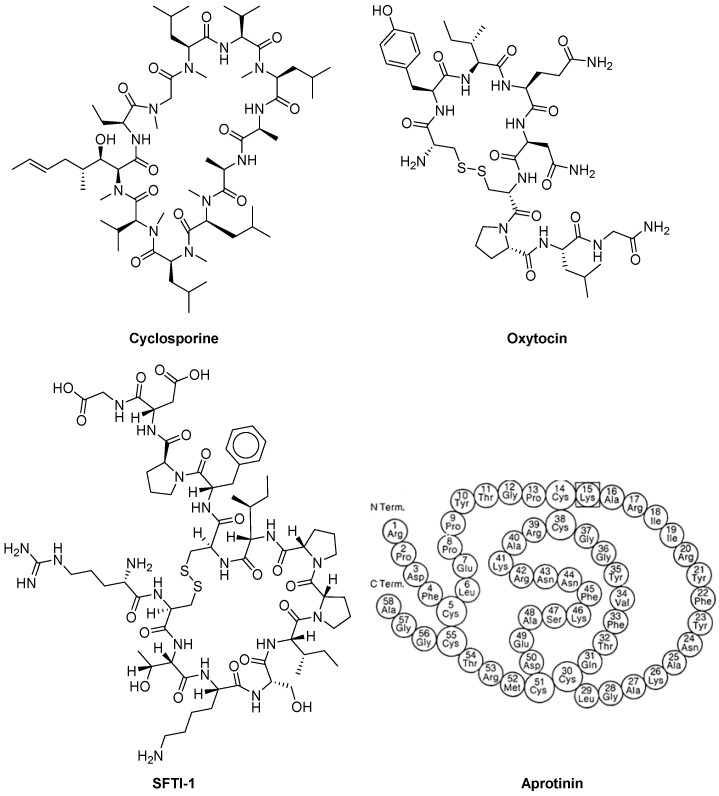
Natural macrocyclic polypeptides.

It is known that the numerous natural MCPPs provide important functions in various hosts, including plants, mushrooms, bacteria, and animals. Many of the natural MCPPs are being sapled as food additives, preservatives, or drugs. Examples of beneficial MCPSs are cyclosporine [178], oxytocin [179], sunflower trypsin inhibitor-1 (SFTI-1) [169,170], and aprotinin [180,181] (Figure 9).

Cyclosporin (CsA) is an MCPP that includes 11 amino acids. It was discovered in 1970 as a natural product of earthen mushrooms. It is stable and has a powerful immunosuppressive activity, which was discovered in 1972 and revolutionized organ transplantation. CsA chemical structure was elucidated in 1976 [182,183]. For the first time in 1983, the FDA approved CsA for clinical use in the United States [184]. Currently, CsA (brands: Sandimmune, Neoral, and Gengraf) is used to treat rheumatoid arthritis, psoriasis, crown disease, nephrotic syndrome, and eczema, as well as to prevent rejection during transplantation of organs [185].

Oxytocin (Oxt) is a small macrocyclic polypeptide (MCPP) composed of nine amino acids, featuring a six-amino acid ring formed by cysteine bonds and a three-amino acid tail ending in a terminal amine. This hormone is produced in key brain regions responsible for maintaining behavioral and physiological equilibrium. In mammals, oxytocin acts as a neurohormone with primary roles in triggering uterine contractions during childbirth, facilitating milk ejection during breastfeeding, and promoting maternal caregiving behavior. Beyond these functions, oxytocin is believed to impact a variety of physiological and behavioral processes, particularly those related to sexual and social interactions in both males and females. In both genders, oxytocin is synthesized in the hypothalamus and then stored and released into the bloodstream by the posterior pituitary gland. It is also produced and secreted in other tissues, including the brain, uterus, placenta, ovaries, and testes [179,186]. Oxt is shown and approved by the FDA in obstetrics to enhance uterine contractions in order to successful vaginal genera of the fetus. In the postpartum period, Oxt is approved for stopping postpartum bleeding and stimulating the postpartum emission of milk. Additional off-label uses of exogenous oxytocin include treating delayed orgasm, inducing sexual arousal, and managing symptoms of autism. Since women distinguish Oxt during intimate interactions, it is believed that it plays a role in social behavior and the formation of communication. [179].

Sunflower trypsin inhibitor-1 (SFTI-1) was isolated in 1999 by Luckett et al. from sunflower seeds [187]. It is one of the most potent trypsin inhibitors of all naturally occurring peptides and belongs to the Bowman–Birk inhibitor (BBI) family. The SFTI-1 structure is a circular peptide of 14 amino acids (~1.5 kDa) containing one disulfide bond that divides the peptide into two loops, one of which is functional trypsin inhibitory loop with a subnanomolar K_i_ value, and the second loop is non-functional.

SFTI-1 is considered a very attractive template for designing engineered proteinase inhibitors, including potent furin inhibitors [188,189], with potential use as pharmacological agents [190,191,192].

SFTI-1 is one of the most popular starting structures for the production of potent and selective inhibitors of a wide range of biologically significant proteases [193]. In particular, native SFTI-1 (&1GRC(&2)TKSIPPIC(&2)FPD&1) has a K_i_ value of 100 pM for trypsin [194], and its synthetic monocyclic analogues exhibit K_i_ values ranging from 1.7 pM to 500 pM [104,108,109] for different proteases (Table 7).

Note that the cyclizations in the sequences presented above are indicated by &, &1, and &2, according to the recommendation of Spengler et al. [199], and changed positions compared to SFTI-1 are shown in bold [193].

Aprotinin (APR) was discovered in 1930 as an “inactivator” of kallikrein in bovine lymph nodes and later, in 1936, as an inhibitor of bovine pancreatic trypsin [204]. APR is a monomeric globular macrocyclic polypeptide (MCPP) with a molecular weight of 6512 Da. It comprises 16 different types of amino acids arranged in a 58-residue chain [205,206], which folds into a stable, compact tertiary structure featuring three disulfide bonds, a twisted β-hairpin, and a C-terminal α-helix. The amino acid sequence for bovine BPTI is RPDFC LEPPY TGPCK ARIIR YFYNA KAGLC QTFVY GGCRA KRNNF KSAED CMRTC GGA. The protein is strongly basic, containing ten positively charged lysine (K) and arginine (R) side chains and only four negatively charged aspartate (D) and glutamate (E) side chains. This basic nature is highlighted in the molecule’s name. The high stability of APR is attributed to its three disulfide bonds, which link the six cysteine residues in the chain (Cys5-Cys55, Cys14-Cys38, and Cys30-Cys51) [181]. The extended, basic side chain of lysine 15 on the exposed loop binds tightly to the specificity pocket at the active site of trypsin, thereby inhibiting its enzymatic activity.

APR is a typical “magic gun” [204,207]—a competitive nanomolar inhibitor of Panproteases (Table 8). It inhibits triprapsin, chymotrypsin, and plasmin in a concentration of about 125,000 IE/mL (IU/ML)) and kallikrein in a concentration of 300,000 IE/mL. Its effect on Kallikrein leads to inhibiting the formation of factor XIIA. As a result, both the internal path of coagulation and fibrinolysis are inhibited. APR on plasmine independently slows down fibrinolysis [205,208]. In addition, APR inhibits the effect of nitrogen oxide synthase types I and II and violates the transport of K^+^ via Ca^2+^-Activated K+ channels [205]. APR inhibits the serine flavivirus protease NS2B-NS3, which breaks down the RNK viral polyprotein [209,210].

APR inhibits TMPRSS2, which is responsible for splitting and activating the SARS-CoV-2 SRS-protein, and thus inhibits the penetration of SARS-CoV-2 into cells [170,211]. APR also inhibits furin and reduces the activity of furin by the SARS-CoV-2 site [212].

APR is an effective anti-inflammatory drug [213,214,215,216], which is called “anti-fibrinolisin of a wide range of action” due to its anti-inflammatory and endothelial-modulating effects. It has many actions that can suppress inflammatory reactions, including weakening platelet activation, maintaining platelet function, reducing complement activation, inhibiting kallikrein products [217], reducing the release of TNF-α [218], IL-6, and IL-8 [217], inhibiting the production of inducible iNOS [219], decreases CPB-induced activation of leukocytes [217,220], and inhibiting the activation of adhesion molecules on monocytes and granulocytes [221,222]. This can reduce the damage to the lungs, reduce the inflammation of the bronchi [223], and weaken the reperfusion damage to the lungs [224].

APR is an inhibitor of serine proteases of the owner, which breaks down the glycoprotein hemagglutinin (on) of the Grtepp virus (IV) and, thus, reduces the replication of the virus. IV cannot initiate an infection of host cells if it is not broken down proteolytically [225]. The subunits of N1 and 2 are much more contagious than their predecessors [226]. APR inhibits the transmembrane serine protease S2 (TMPRSS2), which is necessary for the proteolytic activity.

**Table 8:** Inhibition constants K_i_ for the complexes between APR and various enzymes [227].

Enzyme-Source-Condition	K_i_
Kallikrein (pancreatic, porcine), pH 8.0	1.0 nM
Kallikrein (submandibular, porcine), pH 9.0	1.6 nM
Kallikrein (plasma), pH 8	30.0 nM
Kallikrein (plasma), pH 7.8	100.0 nM
Kallikrein (tissue)	0.8 nM; 1.0 nM
Kallikrein (urine, porcine), pH 9.0	1.7 nM
Kallikrein (urine, human), pH 8.0	0.1 nM
Trypsin (bovine), pH 8.0	0.06 pM
Anhydrotrypsin (bovine), pH 8.0	<0.3 pM
Trypsinogen (bovine), pH 8.0	1.8 μM
Chemotrypsin (bovine), pH 8.0	9.0 nM
Chemotrypsin (bovine), pH 7.0	9.0 nM
Chemotrypsinogen (bovine), pH 8.0	9.0 nM
Plasmin (porcine), pH 7.8	4.0 nM
Plasmin (human), pH 7.8	0.23 nM

##### Engineering of MCPP Furin Inhibitors

Fittler et al., using rational design, constructed new furin inhibitors, including 11 (H-KRCKKSIPPICF-NH_2_), with an inhibition constant K_i_ = 0.49 nM. This furin inhibitor 11 showed weak inhibition of matriptase and trypsin, with K_i_ values of 560 and >10,000, respectively [189]. The researchers selected the SFTI scaffold as their foundation for creating new furin inhibitors due to its constrained structure, ease of synthesis, and bioactivity that directly mirrors alterations in the peptide backbone [203]. They utilized two scaffolds in their design: the monocyclic peptide SFTI-1 [2,25] and the engineered variant SDMI-3 [197]. The SFTI-1 scaffold showed a Ki of 3.5 µM against furin, while SDMI-3, with its Ki of 24 nM, proved to be a promising starting point for further optimization (Table 9). Compared to SFTI-1, the SDMI-3 variant included three key substitutions: Gly1Lys in the canonical loop, as well as Ile10Arg and Phe12His in the C-terminal β-strand.

According to the authors, selective furin inhibitor 11 is a promising compound for the further development of furin inhibitors aimed at controlling the activity of this protease in vitro and in vivo [186].

The high selectivity of picomolar MCPPs 11 and 12 (Table 10) for furin was demonstrated by the authors in assays with matriptase-1 (K_i_ = 0.56 and 4.3 μM, respectively) and trypsin (K_i_ > 10 μM for 11 and 12). This finding is consistent with previous studies by these authors, which showed the importance of residue 12 for interaction with matriptase-1 [229].

The authors also studied the role of the structure-stabilizing disulfide bridge by replacing cysteines with alanines (Table 9, linear peptide **15**). This dramatically reduced its potency (K_i_ = 2.3 μM). The authors also replaced the disulfide bond with 1,4- and 1,5-disubstituted 1,2,3-triazole bridges between positions 3 and 11 of the MCPP and obtained (Table 10) nanomolar MCPPs 16 (K_i_ = 21.8 nM) and 17 (K_i_ = 5.0 nM), respectively. In addition, inhibitors **16** and **17** demonstrated selectivity for furin, as indicated by significantly higher K_i_ values observed in inhibition assays with matriptase-1 and trypsin (Table 10). Although the difference in potency between the two variants for furin inhibition was not as dramatic as that observed for trypsin [203], inhibitor **17** is considered by the authors to be a promising candidate with redox stability for future in vitro and in vivo experiments.

**Figure 10:**
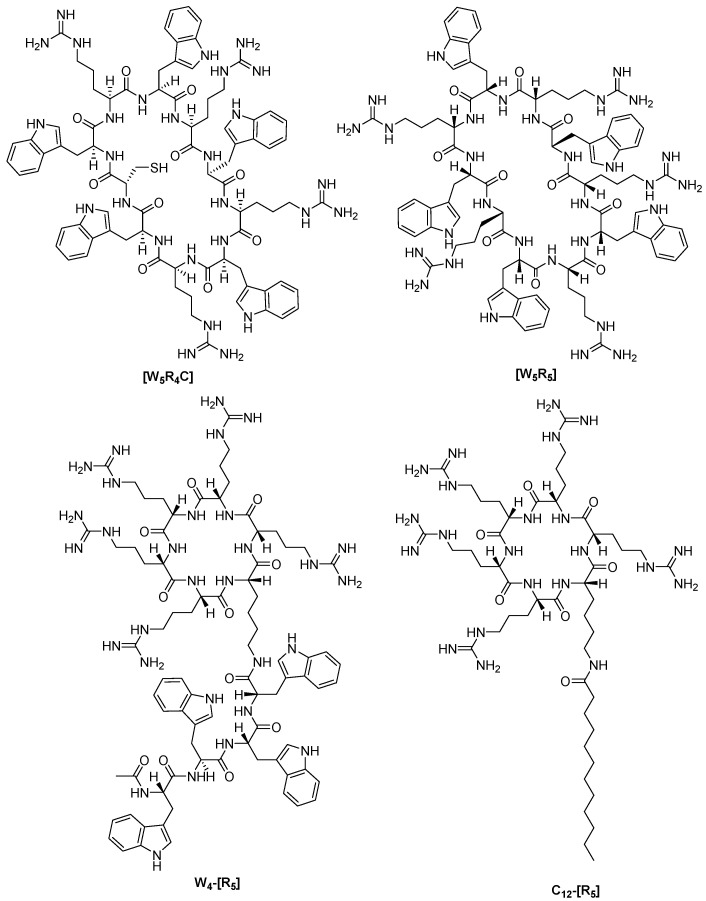
Chemical structures of the polyarginine MCPP furin inhibitors [W5R4C], [W5R5], W4-[R5], and C12-[R5] with K_i_ values 0.34, 0.98, 0.10, and 1.02 μM, respectively.

Ramos-Molina et al. investigated the inhibition of furin activity by polyarginine MCPPs used for intracellular delivery of proteins and drugs. As a result, it was found that MCPPs (Figure 10) inhibit furin in vitro with K_i_ values in the range from 0.1 to 1.02 μM, while the authors did not detect cytotoxicity after 24-h incubation of CHO cells with [WR]5 and W4-[R5] at a concentration of 1 μM [231].

In summary, the authors also highlight that cyclic polyarginine peptides, commonly employed as protein transduction agents, can notably inhibit cellular convertase activity, especially furin. Although this is not necessarily detrimental, for example, in anticancer applications [231], this off-target effect should be taken into account in therapeutic applications of in vivo cell-penetrating peptides containing polyarginines.

Lepek et al. investigated whether different cyclization strategies for ML and ML-Amba octapeptides targeting furin and PACE4 could improve their stability profile [232]. The authors showed that cyclization within a multi-Leu core in combination with the incorporation of a C-terminal 4-amidinobenzylamide (Amba) residue yielded nanomolar macrocyclic inhibitors. The best inhibitors from this group are (&)[Mpa]LLLC(&)RVK[Amba] with K_i_ value 12 and 35 nM for furin and PACE4, respectively, and (&trans-2-butene)[MPA]LLLC(&) RVK[Amba] with the K_i_ value 22 and 26 nM for furin and PACE4, respectively (where & indicates cyclization, and Mpa—3-mercaptopropionic acid). In addition, these inhibitors demonstrated potent inhibitory effects against prostate cancer cell lines as well as improved stability. The authors believe that this cyclic framework could be further used to design even more potent and stable inhibitors.

Linear inhibitors, including MI-1148 [161] and MI-1554 [163], have shown significant activity against numerous furin-dependent pathogenic viruses in cell culture experiments at low micromolar concentrations [28,161,162,163]. However, due to the limited tolerability of the most potent compounds in mice [163], Lam van et al. developed different types of MCPP furin inhibitors (Table 11) and showed that, despite structural differences in the linker segments used and the resulting ring sizes of the inhibitors, they possess closely related P6/P5-P1 segments. For some inhibitors, the authors identified a complete binding mode in complex with furin, which revealed interactions in the active site cleft [233] similar to those of the linear inhibitor H-Arg-Arg-Arg-Val-Arg-4-Amba (K_i_ = 33.7 pM) [234]. Most of the new macrocyclic furin inhibitors have comparable K_i_ values in the range of 0.5–1 nM (Table 10) [233].

However, all newly synthesized macrocyclic derivatives, including the most potent inhibitor 10 containing the cyclic CPP segment, exhibited minimal antiviral activity in RSV-infected cells, whereas the linear reference inhibitors MI-1148 [161] and MI-1554 [163] significantly reduced viral replication at low micromolar concentrations.

The authors suggest that the lack of antiviral activity observed in the cyclic analogues may be due to an insufficient amount of these inhibitors reaching furin in the secretory pathway where F0 processing occurs, likely because of their increased molecular weight. Another possible reason for the lack of antiviral activity could be the reduced inhibitory potency of the cyclic derivatives compared to the highly potent picomolar inhibitors MI-1148 and MI-1554.

Recently, Gitlin-Domagalska et al. [107] developed new, strong, and stable mono- or bimacrocyclic peptide furin inhibitors based on the structure of the extensively studied trypsin inhibitor SFTI-1 through combinatorial chemistry. These inhibitors exhibit K_i_ values in the subnanomolar range (Table 12). Inhibitor 5, with K_i_ = 0.21 nM, was found to be the most active and significantly more proteolytically resistant than the reference furin inhibitor FI [189] described in the literature. Additionally, it decreased furin-like activity in PANC-1 cell lysate.

### 2.2. Non-Covalent Small-Molecule Furin Inhibitors

The strong furin inhibitors developed previously and presented above are peptide derivatives or peptidomimetics containing strongly basic amidine and/or guanidine moiety residues that provide them with high inhibitory activity. In this regard, the first small-molecule non-covalent furin inhibitors included several guanidine moieties, including guanidinylated aryl-2,5-dideoxystreptamines (GADDs), published in 2006 [231,235]. Some of the GADDs have demonstrated potent inhibitory activity against furin in biochemical and cellular assays. Among the studied series of GADDs, the most active inhibitors of furin were nanomolar GADDs 1e and 1g, which were found to behave as competitive inhibitors of furin and to be relatively specific for furin. (Table 13). GADDs have a higher selectivity for furin compared to non-PC enzymes such as trypsin, lactoferrin (LF), and matrix metalloproteinase (MT1-MMP). GADDs show a preference for furin and PC6B over PACE4 and PC7. In a control experiment, in the absence of furin and FP59 in cell assay buffer, GADDs did not exhibit significant cytotoxicity to cells at concentrations up to 250 μM. GADDs inhibit furin-dependent anthrax PA processing in a cell-based assay, demonstrating their therapeutic potential. Additionally, control experiments showed no detectable cytotoxicity. According to the authors, GADDs may not only serve as valuable tools for studying the action of furin but also have therapeutic applications as short-acting antiviral and antibacterial agents [235].

Dahms et al., as a result of X-ray diffraction studies, discovered new binding modes of the inhibitor with furin. They found that furin complexed with GADD **1n** (Table 12) involved two **1n** molecules interacting with furin. One molecule is securely anchored in the S4 pocket, directly disrupting the conformation and function of the catalytic triad, while the other molecule exhibits weaker binding and interacts with a more distant, less conserved region of furin. The authors suggest that these newly identified binding modes represent a fresh approach to furin inhibition and offer the potential for achieving specificity among proprotein convertases (PCs). This discovery provides an innovative foundation for the structure-guided development of furin inhibitors [236].

**Figure 11:**
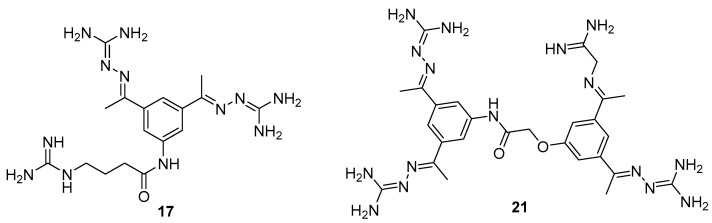
Small-molecule furin inhibitors **17** (K_i_ = 0.46 µM) and **21** (K_i_ = 0.59 µM) [237].

It should be noted that the chemical and pharmacokinetic properties of very highly basic polypeptide and peptidomimetic inhibitors limit their use as therapeutic agents. In this regard, some efforts have been made to search for small-molecule furin inhibitors with reduced basicity. As a result of these efforts, various micromolar furin inhibitors were obtained [237,238,239], among which guanylhydrazones (GG) **17** (K_i_ = 0.46 μM) and **21** (K_i_ = 0.59 μM) emerged as the most active low-molecular-weight furin inhibitors (Figure 11) [237].

X-ray diffraction studies of furin complexes with model GGs showed that the latter interact uniquely within the S1 pocket, which differs significantly from substrate-like ligands. A second binding site has been identified in the S4/S5 pocket of furin; however, the S1 site is the primary binding pocket [240].

**Figure 12:**
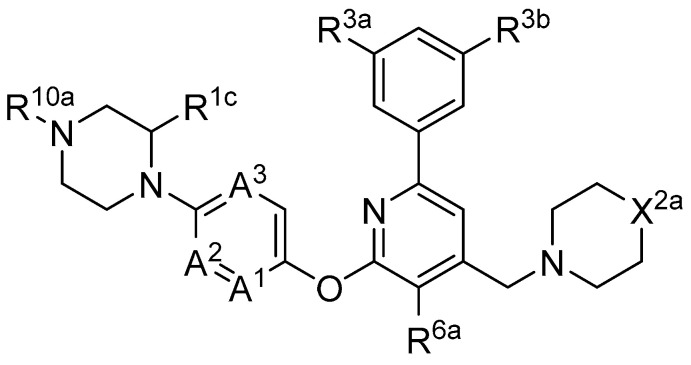
Furin inhibitors from WO/2019/215341. A^1^, A^2^, and A^3^ are each independently N or CH, wherein one or two of A^1^, A^2^, and A^3^ are N; X^2a^ is NR^10b^ or C(R^11a^)R^12a^; R^1c^ is hydrogen; R^3a^ and R^3b^ are each independently F or Cl; R^6a^ is H, F, Cl, or Me; R^10a^ is hydrogen, (C_1_-C_4_)alkyl, or (C_3_-C_6_)cycloalkyl, which is optionally substituted by -CO_2_H, -CONH_2_, -CONH(C_1_-C_4_)alkyl, -CON((C_1_-C_4_)alkyl)((C_1_-C_4_)alkyl), OH, O(C_1_-C_4_)alkyl, -SO_2_(C_1_-C_4_)alkyl, or -SO_2_NH_2_; or R^10a^ and R^1c^ taken together represent -CH_2_- or -(CH_2_)_2_-; R^10b^ is (C_1_-C_4_)alkyl, which is optionally substituted by -CONH_2_, -CONH(C_1_-C_4_)alkyl, or -CON((C_1_-C_4_)alkyl)((C_1_-C_4_)alkyl); R^11a^ is (C_1_-C_4_)alkyl or (C_1_-C_4_)alkoxy, each of which is optionally substituted by one or two substituents independently selected from -CO_2_H, -CONH_2_, -CONH(C_1_-C_4_)alkyl, -CON((C_1_-C_4_)alkyl)((C_1_-C_4_)alkyl), OH, -OCONH(C_1_-C_4_)alkyl, -NHCO(C_1_-C_4_)alkyl, -NHCO_2_(C_1_-C_4_)alkyl, and -NHCONH(C_1_-C_4_)alkyl; R^12a^ is H, OH, or F. When R^12a^ is OH, R^11a^ is (C_1_-C_4_)alkyl, which is optionally substituted by one or two substituents independently selected from -CO_2_H, -CONH_2_, -CONH(C_1_-C_4_)alkyl, -CON((C_1_-C_4_)alkyl)((C_1_-C_4_)alkyl), OH, -OCONH(C_1_-C_4_)alkyl, -NHCO(C_1_-C_4_)alkyl, -NHCO_2_(C_1_-C_4_)alkyl, and -NHCONH(C1-C_4_)alkyl; and m is 1 or 2.

In 2019, Axten et al. developed and patented furin inhibitors, specifically hundreds of compounds with the general formula III (Figure 12), for use in the treatment of fibrotic diseases, including pulmonary fibrosis, renal fibrosis, liver fibrosis, skin fibrosis, ocular fibrosis, cardiac fibrosis, and other various fibrotic conditions. According to the authors, these furin inhibitors may also be useful for the treatment of other furin-mediated conditions, including but not limited to hypertension, cancer, infectious diseases, genetic disorders (e.g., cystic fibrosis), and neurodegenerative disorders [241].

The most active in the studied series of compounds were the picomolar furin inhibitors **1**–**3**, and the most attractive were compounds **4**–**8** (Table 14), which later attracted increased attention [105,242].

The substitution pattern of (3,5-dichlorophenyl)-pyridine-based inhibitors has a decisive influence on their potency and pharmacological properties. Comparatively high bioavailability of **2**, **3**, and **4** was observed in a mouse model of bleomycin-induced pulmonary fibrosis. Total TGFβ production in the lungs was reduced by 75%, 86%, and 69% for **2**, **3**, and **4** at a dose of 10 mg/kg body weight, respectively. Compound **2** was administered orally to mice, whereas compounds **3** and **4** were administered intraperitoneally.

Douglas et al. found that BOS-318 is an exceptionally potent and highly selective furin inhibitor, with an IC_50_ value of 1.9 ± 1.1 nM (Table 14) [105]. Beyond just assessing its potency, they conducted an in-depth kinetic analysis using a peptide substrate, which revealed an inhibitor constant (K_i_) of 0.413 nM. When tested against other closely related proprotein convertase subtilisin/kexin-type proteases (PCSK), BOS-318 was found to be about 13 times more potent against furin than PCSK5, 24 times more potent than PCSK7, and 110 times more potent than PCSK6 (Table 14). This selectivity is superior to that of the commonly used furin inhibitor dec-RVKR-CMK, which exhibited similar potency across all PCs tested (Table 15).

Furthermore, BOS-318 demonstrated cell permeability and effectively localized to the Golgi compartment. Its EC50 against endogenous proteases in the Golgi was 23.5 ± 14.7 nM (Table 14), showing only a 12-fold decrease in potency compared to its biochemical activity (IC_50_ = 1.9 ± 1.1 nM). In stark contrast, the less permeable dec-RVKR-CMK exhibited a dramatic 7000-fold decrease in cellular potency (EC_50_ = 9108 ± 6187 nM). These findings underscore BOS-318’s adequate permeability and potency, making it a valuable tool for the pharmacological evaluation of intracellular furin activity [105].

A broad screening of BOS-318 at a 10 mM concentration against a panel of 64 serine, cysteine, and matrix metalloproteinases revealed minimal off-target effects, with 61 proteases showing either no inhibition or less than 20% inhibition. Additionally, BOS-318 did not inhibit proteases involved in the activation of ENaC, such as prostasin, NE, cathepsin B, cathepsin S, chymotrypsin, matriptase, and plasmin. The highest observed inhibition under these conditions was approximately 30%, noted for chymase [105].

BOS-318 was also found to mitigate PEA-induced epithelial cell toxicity and significantly reduced ENaC activity, which increased airway surface liquid (ASL) height and mucociliary clearance (MCC) rates in fully differentiated cystic fibrosis (CF) primary human bronchial epithelial cells (HBECs). These results strongly support furin inhibition as a therapeutic strategy for CF lung disease, positioning highly selective compounds like BOS-318 as promising candidates for future drug development [105,244].

BOS-318 and its analogues feature a novel core structure, 2-(3,5-dichlorophenyl)-pyridine, which is less charged compared to traditional inhibitors that utilize the polybasic furin consensus motif. This innovation results in inhibitors with a distinct mode of furin binding, characterized by a reorganization of the substrate binding site. This reorganization exposes a new hydrophobic pocket that accommodates the dichlorophenyl moiety of BOS-318, which engages in a unique network of interactions without directly involving the catalytic triad residues D153, H194, or S368. BOS-318’s cell permeability, exceptional selectivity for furin, and efficacy in treating CF symptoms in an ex vivo human model make it a promising therapeutic candidate [105,241]. BOS-318 notably reduces ENaC-mediated sodium absorption and protects against neutrophil elastase activation of ENaC in fully differentiated primary human bronchial CF cells. Additionally, it increases ASL height, enhances MCC clearance rates, and provides cytoprotection against P. aeruginosa furin-activated exotoxin A-induced airway epithelial cell toxicity, a critical factor in CF lung disease [105]. Overall, BOS-318 shows great promise as a potential therapeutic for CF, with future studies needed to assess its efficacy and safety in animal models and beyond.

Soon after the work of Douglas et al. [105], Essalmani et al. published the results of studies on the activity of BOS-318 and two other furin inhibitors, BOS-857 and BOS-981, the structure of which was not disclosed [31]. Non-toxic, cell-penetrating inhibitors are available for both oral (BOS-981 and BOS-318) and inhaled (BOS-857) administration. The authors assessed their in vitro potency and selectivity on purified forms of furin, PC5A, PACE4, and PC7 (Table 16). The inhibitors effectively blocked substrate processing by all convertases, with IC_50_ values ranging from ∼7 to 9 nM, compared to ∼9 to 10 nM for the known cell-permeative PC inhibitor dec-RVKR-cmk. The BOS inhibitors also effectively inhibit the cleavage of SARS-CoV-2 S1/S2 by furin, with IC_50_ values of 4 ± 0.7 nM, 32 ± 4 nM, and 35 ± 5 nM for BOS-981, BOS-857 and BOS-318, respectively [31].

The researchers were the first to demonstrate that small-molecule non-peptide BOS inhibitors can effectively block SARS-CoV-2 entry by targeting furin and preventing viral fusion at the cell surface through a pH-independent pathway. This inhibitory effect was notably enhanced when combined with TMPRSS2 inhibitors. BOS inhibitors successfully blocked the processing of the spike (S) protein within HeLa cells, and the combined use of furin (BOS) and TMPRSS2 (Camostat) inhibitors completely prevented SARS-CoV-2 infection in Calu-3 lung cells. Detailed analyses of cell–cell fusion and S protein processing revealed that ACE2 shedding by TMPRSS2 is crucial for enhancing fusion in the absence of S1/S2 priming. Furthermore, this study highlighted the importance of the dimerization domain of the collectrin region of ACE2 in TMPRSS2-mediated cell fusion. These findings underscore the synergistic role of furin and TMPRSS2 in facilitating viral entry and infection, suggesting that a combination of furin and TMPRSS2 inhibitors could serve as a powerful antiviral strategy against SARS-CoV-2. The non-toxic BOS furin inhibitor, when used in conjunction with a TMPRSS2 inhibitor, significantly reduced viral entry into lung cells, achieving an approximate 95% reduction in viral infection—a promising approach for combating the spread of SARS-CoV-2, including Omicron variants [29,31].

Shortly after the publication of Douglas et al. [105] and Essalmani et al. [31], Dahms et al. revealed the crystal structures of several BOS inhibitors (compounds **5**–**9**, listed in Table 13), which are now accessible in the Protein Data Bank (PDB) under IDs 7QY0, 7QY2, 7QXY, 7QY1, and 7QXZ. The binding mechanisms of these inhibitors to furin were found to be consistent with the previously reported mechanism for BOS-318. These dichlorophenyl (DCP)-pyridine “BOS” drugs, including BOS-318, inhibit human furin through a competitive binding process that involves an induced-fit mechanism. In this process, the tryptophan residue W254 within furin’s catalytic cleft acts as a molecular gate, rotating nearly 180° to reveal a hidden hydrophobic pocket. The nonpolar DCP group of BOS-318, along with similar halogenated phenyl groups in related compounds, binds within this cryptic pocket, stabilizing the drug’s attachment.

However, computational results presented in communication by Ridgway et al. [245] suggested that current flexible-receptor docking methods were unable to replicate this induced-fit mechanism for known furin inhibitors like BOS-318. Even for BOS-318 analogues, such as Mod23 with a terminal ethyl-tetrazole group, the docking energies showed only moderate improvement over the parent compound.

Mutational analysis in this study indicated that variants like W254G (with a minimal hydrogen side chain) or W254F (in the open conformation) allowed BOS ligands to access the cryptic pocket. Docked poses showed that the indole and phenyl sidechains of W254 and W254F engaged in hydrophobic, π–π resonance, and ionic interactions with most BOS ligands. Interactive molecular dynamics (iMD) simulations further identified two potential pathways for BOS ligand entry into the furin catalytic cleft, coupled with W254 dihedral rotation and the opening of the cryptic pocket. The simulations also suggested that energy fluxes from disrupting and reforming solvation shells during the transition from solution to the furin catalytic cleft might drive BOS ligand entry and binding [245].

Dahms et al. also developed a furin activity assay based on MALDI-TOF-MS and determined IC_50_ values for these compounds using peptide substrates derived from TGFβ and the S protein of SARS-CoV-2. Under the conditions studied, inhibitors **5**–**9** showed nanomolar activity, which in the case of the TGFβ substrate, showed IC_50_ values of 2.3, 1.3, 1.8, 2.6, and 78 nM for compounds **1**–**5**, respectively. When using the S protein substrate, the authors observed IC_50_ values of 1.1 and 0.8 nM for compounds **1** and **2**, respectively [105]. Based on published data [241], the authors also noted that the nature of substitution on inhibitors based on 2-(3,5-dichlorophenyl)-pyridine decisively affects their effectiveness and pharmacological properties. In particular, the comparatively high bioavailability of inhibitors **6**, **7**, and **8** was reported in a mouse model of bleomycin-induced pulmonary fibrosis, with total pulmonary TGFβ production reduced by 75%, 86%, and 69% for inhibitors **8**, **7**, and **6** at a 10 mg dose/kg body weight, respectively. Inhibitor **6** was given to mice orally, whereas inhibitors **7** and **8** were administered intraperitoneally.

The obtained data support the use of furin inhibitors as a future therapy for Exo-A-induced lung injury and mortality in PA infection [243] and, as shown above, reducing the incidence of virus infection [29,31].

## 3. Toxicity of Non-Covalent Furin Inhibitors in Mice Models

The results of toxicity studies on picomolar non-covalent polypeptide furin inhibitors in mice are presented in Table 17 [167,172]. As can be seen from this table, the toxicity of this type of compound does not correlate with their antiviral activity. In contrast to the control picomolar inhibitors 1 and 2, reduced toxicity was found for all Cav derivatives **5**, **6**, **8**, and **17**.

Similar plasma levels were obtained after IV treatment of rats with 1 mg/kg compounds **1** and **8**. However, two of the three rats died within 90 min of intravenous treatment with inhibitor **1**, whereas compound **8** showed reduced toxicity in all three rats without any signs of adverse effects [167].

It should be noted that compounds **8** and **17** differ in the P1 group but have similar potency and low toxicity. This suggests that the P1 of the Amia (3-amino-1H-isoindol-6-yl) group has a similar effect on the toxicity profile of these furin substrate analogue inhibitors in mice as the Amba group.

## 4. Efficiency of Non-Covalent Furin Inhibitors in Mice Models

Positive results from a number of studies on the effectiveness of furin inhibitors in mouse models have been published, including the effectiveness of treatment of murine pulmonary fibrosis induced by bleomycin [241], acute lung injury in a mouse model of Pseudomonas Aeruginosa infection [243], colorectal cancer [246], vascular remodeling and coronary atherosclerosis [70], epilepsy [246], and the regulation of learning and memory [155].

Data on the efficacy of furin inhibitors in the treatment of murine pulmonary fibrosis induced by a single intrapulmonary administration of bleomycin have been reported [241]. The efficacy of the drugs was assessed by inhibiting TGFβ secretion and collagen deposition in the mouse lungs. The day before bleomycin administration, the compounds were administered to mice depending on the appropriate route and frequency. On the day of the study initiation, all animals were anesthetized with ketamine (80 mg/kg) and xylazine (10 mg/kg) and were cannulated orotracheally and injected intratracheally with 50 mL of saline (control) or 0.03 U bleomycin in 50 mL of saline (all groups except the control). The drugs were administered daily until the end of the study on day 15. On the day of termination, the animals were euthanized by CO_2_ inhalation. The right and left lungs were collected and analyzed for TGFβ and hydroxyproline after appropriate processing. The results obtained are presented in Table 18 and indicate the in vivo effectiveness of the tested drugs in the treatment of pulmonary fibrosis in mice.

Recently, Bernard reported the results of a study of the efficacy of BOS-318 (comp. 4 with IC_50_ = 0.8 nM, or comp. 207, pIC_50_ = 9.1 [241]) in treating acute lung injury in a mouse model of Pseudomonas Aeruginosa infection [243]. In this study, 10–12-week-old B6 WT mice were treated with 5 mM BOS-318 or vehicle (control) via intraperitoneal injection one hour before the intra-oral administration of 250 ng of PA Exo-A, with or without BOS-318. The inhibitor was subsequently injected intraperitoneally at 24, 48, and 72 h. A survival study was conducted over a period of 10 days. Inoculation of Exo-A caused 66% death by day 6 and 80% by day 8 in the control group, while all mice treated with BOS-318 survived and were euthermic by day 4. Mice treated with BOS-318 were significantly less hypothermic at 24 h and lost less weight at 72 h compared to control. Neutrophil counts were lower in the BOS-318 group at 48 h compared to the control group, with an increase in monocytes in the BALF, indicating reduced lung inflammation. Blood neutrophil counts were also lower at 72 h, while platelet counts were higher in the BOS-318 group. BALF levels of MIP-2, KC, TNF-a, PF4, platelets, monocytes, and NETs were higher in mice treated with BOS-318 at 72 h, whereas protein concentrations were lower compared to the control group. The authors suggest that these findings support the potential of the furin inhibitor BOS-318 as a future therapy for Exo-A-induced lung injury and mortality in PA infection. The authors suggest that BOS-318 decreases neutrophilic lung inflammation induced by Exo-A and report that they are continuing further studies to understand whether this pathway is mediated by platelets or other cytokines [243].

He et al. demonstrated for the first time that genetic inactivation of furin suppresses tumor development, proliferation, and migration in colorectal cancer (CRC) cell lines with KRAS or BRAF mutations but not in cells with wild-type KRAS or BRAF. Given that mutations in KRAS and BRAF, which activate the ERK kinase pathway, are common in CRC and contribute to resistance against targeted therapies, the researchers investigated a mouse xenograft model. They found that KRAS or BRAF mutant cells lacking furin exhibited reduced growth, decreased angiogenesis, and increased apoptosis. Mechanistically, furin inactivation blocked the processing of several protein precursors, including proIGF1R, proIR, pro-cMET, proTGF-β1, and NOTCH1, leading to potent and sustained suppression of the ERK-MAPK pathway in mutant cells. Additionally, genes involved in activating the ERK-MAPK pathway, such as PTGS2, were downregulated in mutant cells after furin inactivation but upregulated in wild-type cells. Analysis of human colorectal tumor samples showed a positive correlation between elevated furin expression and KRAS or BRAF mutations. The authors suggest that furin plays a crucial role in the activation of the ERK-MAPK pathway and tumorigenesis in KRAS or BRAF mutant cancers, presenting a potential target for personalized therapy [246].

Yakala et al. explored the impact of systemic furin inhibition on vascular remodeling and coronary atherosclerosis in hyperlipidemic Ldlr−/− mice using the irreversible furin inhibitor α-1-PDX (α1-antitrypsin Portland). They discovered that in vivo administration of α-1-PDX to these mice resulted in a significant reduction in atherosclerotic lesion area, particularly in severe lesions. This was accompanied by a decrease in lesional macrophage and collagen content, as well as lower levels of systemic inflammatory markers. Matrix metallopeptidase 2 (MMP2), a furin substrate involved in endothelial function and atherosclerotic lesion progression, was significantly reduced in the aortas of treated mice [70].

To further investigate furin’s role in vascular endothelial function, Yakala et al. administered α-1-PDX to Apoe−/− mice with wire-induced injury in the common carotid artery. They observed a marked reduction in carotid intimal thickness, plaque cellularity, smooth muscle cell and macrophage content, and inflammatory markers, suggesting that furin inhibition provides protection against vascular remodeling. In this model, overexpression of furin led to a significant 67% increase in intimal plaque thickness, reinforcing the direct correlation between furin levels and atherosclerosis [66]. The authors concluded that systemic furin inhibition reduces vascular remodeling and atherosclerosis, potentially through the modulation of MMP2 activity, offering atheroprotective benefits in hyperlipidemic Ldlr−/− mice [70].

Yakala et al. also studied the role of furin in epilepsy. They found that furin protein levels were elevated in the temporal neocortex of epileptic patients and in the hippocampus and cortex of epileptic mice. Furin transgenic mice exhibited increased susceptibility to epilepsy and heightened epileptic activity compared to wild-type mice, while lentivirus-mediated knockdown of furin reduced epileptic activity. Using whole-cell patch-clamp techniques, the authors demonstrated that furin knockdown and overexpression influenced neuronal inhibition by altering postsynaptic gamma-aminobutyric acid A receptor (GABAAR)-mediated synaptic transmission. Furin modulated the expression of GABAAR β2/3 subunits at the membrane and total protein levels in epileptic mice by affecting transcription rather than protein degradation. These findings suggest that furin regulates GABAAR-mediated inhibitory synaptic transmission by modulating GABAAR β2/3 subunit transcription, providing new insights into epilepsy prevention and treatment [247].

Zhu et al. generated furin transgenic (Furin-Tg) mice using the MoPrP promoter to drive the expression of full-length wild-type (WT) mouse furin and crossed them with C57BL/6J WT mice to establish transgenic lines. In brain-specific Furin-Tg mice, dendritic spine density and neural progenitor cell proliferation were significantly increased. These mice also exhibited enhanced long-term potentiation (LTP) and improved spatial learning and memory without alterations in miniature excitatory or inhibitory postsynaptic currents. In the cortex and hippocampus of Furin-Tg mice, the ratio of mature brain-derived neurotrophic factor (mBDNF) to pro-BDNF and the activities of the extracellular signal-regulated kinase (ERK) and cAMP response element-binding protein (CREB) were significantly elevated. Additionally, hippocampal knockdown of CREB reduced the enhancement of LTP and cognitive function in Furin-Tg mice. The authors concluded that furin promotes dendritic morphogenesis and enhances learning and memory in transgenic mice, likely through the BDNF–ERK–CREB signaling pathway. These findings offer new insights into the molecular mechanisms underlying learning and memory and may inform treatment strategies for neurological disorders such as Alzheimer’s disease [154].

## 5. Conclusions

Furin, the first proprotein convertase (PC) to be identified, is ubiquitous in mammalian cells and plays a crucial role in the maturation of a diverse array of proproteins. The expression and activity of furin are involved in numerous physiological and pathological processes, ranging from embryonic development to the progression of cancer [68]. Since its discovery, furin has become a promising target for the treatment and prevention of infectious diseases that depend on host protease activity for infection. It is well-established that viral surface glycoproteins and bacterial toxins possess paired basic amino acid sequences that are recognized and cleaved by furin and other PCs, with this cleavage often being essential for viral entry and replication. Hallenberger et al. were the first to highlight the therapeutic potential of furin inhibitors in combating viral infections, marking a significant advance in the field [38].

The studies presented in this review demonstrate the development and research of drug candidates for the treatment of infectious and non-infectious diseases in the field of polypeptide furin inhibitors, covalent and non-covalent peptidomimetic furin inhibitors, including macrocyclic peptidomimetics and small molecules. The most active and attractive furin inhibitors with different mechanisms of action were the peptide mimetic MI-1851 and the small molecule BOS-318, with the latter being the most advanced drug candidate, showing in particular efficacy and safety in treating acute lung injury in a mouse model of Pseudomonas Aeruginosa infection [243].

Thus, future researchers should understand and consider the heterogeneous functions of furin when developing effective furin-targeting strategies for the treatment of infectious and non-infectious diseases.

## Figures and Tables

**Table 1:** Inhibition constants of polyarginine peptides for furin, PACE4, and PC1 [151].

Inhibitor	K_i_, μM		
	Furin	PACE4	PC1
Tetra-L-arginine (L4R)	6.4 ± 0.9	>200	>200
Penta-L-arginine (L5R)	0.99 ± 0.08	0.98 ± 0.120	14 ± 6.1
Hexa-L-arginine (L6R)	0.114 ± 0.006	0.52 ± 0.045	3.9 ± 0.62
Hepta-L-arginine (L7R)	0.068 ± 0.001	0.24 ± 0.045	5.2 ± 1.2
Octa-L-arginine (L8R)	0.061 ± 0.001	0.15 ± 0.060	5.1 ± 2.0
Nona-L-arginine (L4R)	0.042 ± 0.003	0.11 ± 0.013	12 ± 2.5
Hexa-D-arginine (D6R)	0.106 ± 0.010	0.58 ± 0.040	13 ± 0.25

**Table 2:** Inhibition of furin by inhibitors of the general formula P5-Arg-Val-P2-P1 [157].

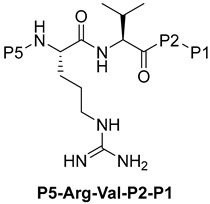				
No.	P5	P2	P1	K_i_, nM
**3**	Phac	Arg	NH_2_(CH_2_)_3_NH	3020
**4**	Phac	Arg	Gua(CH_2_)_3_NH	63
**5**	Phac	Arg	NH_2_(CH_2_)_4_NH	7490
**6**	Phac	Arg	Gua(CH_2_)_4_NH	78
**7**	Phac	Arg	NH_2_(CH_2_)_5_NH	553
**8**	Phac	Arg	Gua(CH_2_)_5_NH	1070
**9**	Phac	Arg	4-NH_2_CH_2_-BnNH	627
**10**	Phac	Arg	4-GuaCH_2_-BnNH	1430
**11**	Phac	Arg	3-NH_2_CH_2_-BnNH	1320
**12**	Phac	Arg	3-GuaCH_2_-BnNH	2730
**13**	Phac	Arg	1H-piperidin-4-yl-CH_2_NH	9710
**14**	Phac	Arg	1-carboximidamide-piperidin-4-yl-CH_2_NH	53
**15**	Phac	Arg	4-amidino-Bn-NH	0.81
**16**	Dec	Arg	4-amidino-Bn-NH	1.6
**17**	Ac	Arg	4-amidino-Bn-NH	1.0
**18**	Dec	Lys	4-amidino-Bn-NH	3.3

Here and further: Phac—phenacyl, Gua—guanidinyl, Bn—benzyl, Dec—CH_3_(CH_2_)_8_C=O, Ac—CH_3_C=O.

**Table 3:** Specificity of selected inhibitors towards furin-like PCs and trypsin-like serine proteases (n.d., not determined) [157].

No.	K_i_, nM								
	Furin	hPACE4	hPC5/6	hPC7	hPC1/3	hPC2	Thrombin	fXa	Plasmin
**6**	78	42	85	>10,000	53	>10,000	102,000	83,000	97,000
**14**	53	67	173	>10,000	70	>10,000	50,000	123,000	>1,000,000
**15**	0.81	0.6	1.6	6154	0.75	312	23,000	40,000	6000
**16**	1.6	3.0	6.3	968	3.65	55	n.d.	n.d.	n.d.
**17**	1.0	2.4	3.6	5131	1.7	1388	n.d.	n.d.	n.d.

**Table 4:** Inhibition of furin by inhibitors of the type P5-P4-P3-P2-4-Amba [158].

No.	P5	P4	P3	P2	K_i_, nM
**2**	Phac	Arg	Ala(Gua)	Arg	0.83
**3**	Phac	Arg	Ile	Arg	0.84
**4**	Phac	Arg	Phe	Arg	1.40
**5**	Phac	Arg	Tyr	Arg	1.9
**6**	Phac	Arg	Arg	Arg	3.0
**7**	Phac	Arg	Dap	Arg	3.0
**8**	Phac	Arg	Lys	Arg	3.6
**9**	Phac	Arg	Cys	Arg	5.9
**10**	Phac	Arg	Tyr	Arg	6
**11**	Phac	Arg	His	Arg	6.3
**12**	Phac	Arg	Trp	Arg	6.3
**13**	Phac	Arg	Met	Arg	9.7
**14**	Phac	Arg	Leu	Arg	12
**15**	Phac	Arg	Gin	Arg	13
**16**	Phac	Arg	Ala	Arg	19
**17**	Phac	Arg	Asn	Arg	23
**18**	Phac	Arg	Ser	Arg	24
**19**	Phac	Arg	Pro	Arg	37
**20**	Phac	Arg	Gly	Arg	40
**21**	Phac	Arg	Glu	Arg	102
**22**	Phac	Arg	Asp	Arg	405
**23**	Phac	Lys	Val	Arg	285
**24**	Phac	Cit	Val	Arg	238
**25**	Phac	Lys(Cbz)	Val	Arg	702
**26**	Phac	Arg	DVal	Arg	1110
**27**	Phac	Arg	DAla	Arg	1385
**28**	Phac	DArg	Val	Arg	970
**29**	Phac	Val	DArg	Arg	7340
**30**	-	Ac	Val	Arg	2390
**31**	-	Phac	DArg	Arg	3200
**32**	Phac	Arg	Val	Nα (Me)Arg	142
**33**	Phac	Arg	Val	Lys	1.5
**34**	Phac	Arg	Dap	Lys	3.7

**Table 5:** Inhibition of furin by inhibitors of the general formula [28].

No.	P5 *	K_i_, nM	IC_50_, nM
**2**	Bn-C=O	7.6	33
**3**	Ac	1.0	n.d.
**4**	Pr-C=O	0.67	n.d.
**5**	Am-C=O	0.78	n.d.
**6**	Hep-C=O	0.67	2.3
**7**	Nonyl-C=O	1.6	8.3
**8**	Undecyl-C=O	5.6	n.d.
**9**	Tridecyl-C=O	50	396
**10**	Pentadecyl-C=O	n.d.	80
**11**	Heptadecyl-C=O	n.d.	14,010
**12**	CH_3_(CH2)_5_-CH=CH-(CH2)_7_-C=O	n.d.	289
**13**	CH_3_(CH2)_7_-CH=CH-(CH2)_7_-C=O	n.d.	272
**14**	CH_3_(CH_2_)_4_-CH=CH-CH_2_-CH=CH-(CH2)_7_-C=O	5.3	22
**15**	3,4-di-Cl-Phac	1.2	n.d.
**16**	Tos	5.3	n.d.
**17**	BnO-C=O	2.4	n.d.
**18**	Fmoc	8.5	n.d.
**19**	H_2_N-(CH_2_)_5_-C=O	0.096	n.d.
**20**	Gua-(CH_2_)_5_-C=O	0.085	n.d.
**21**	H_2_N-(CH_2_)_4_-C=O	0.070	n.d.
**22**	Gua-(CH_2_)_4_-C=O	0.062	n.d.
**23**	4-NH_2_CH_2_-Bn-C=O	0.033	n.d.
**24**	4-GuaCH_2_-Bn-C=O	0.016	n.d.
**25**	3-NH_2_CH_2_-Bn-C=O	0.037	n.d.
**26**	3-GuaCH_2_-Bn-C=O	0.008	n.d.
**27**	2-NH_2_CH_2_-Bn-C=O	0.127	n.d.
**28**	2-GuaCH_2_-Bn-C=O	0.291	n.d.

* Tos –p-toluenesulfonyl; Fmoc—fluorenylmethoxycarbonyl, n.d.—not determined.

**Table 6:** Inhibition of furin-like PCs and thrombin by inhibitors **21**–**26** [28].

No.	K_i_, nM							
	Furin	hPACE4	hPC5/6	hPC7	hPC1/3	hPC2	thrombin	fXa
**21**	70	52	136	69	75	1.2 × 10^5^	>10^6^	2.5 × 10^7^
**22**	62	11	68	13	24	9.1 × 10^4^	>10^6^	1.8 × 10^6^
**23**	33	5 *	24	6.3	30	2.7 × 10^4^	>10^6^	3.6 × 10^7^
**24**	16	5 *	11.2	1.5	17	2.3 × 10^4^	>10^6^	1.3 × 10^7^
**25**	37	44	140	22	74	6.4 × 10^4^	>10^6^	1.1 × 10^7^
**26**	8	1.7	23	2.9	5.1	3.2 × 10^4^	>10^6^	5.9 × 10^6^

* K_i_ values of 5 pM were determined for inhibitors **23** and **24** against hPC1/3 based on a single measurement only due to the limited amount of the enzyme.

**Table 7:** SFTI-1-based selective inhibitors.

Target	Sequence	Ki
Trypsin	&1GRC(&2)TKSIPPIC(&2)FPD&1 (native SFTI-1)	100 pM [194]
Trypsin	GRC(&)TKSIPAIC(&)FPD	1.7 pM [195]
Trypsin	GKC(&)TKSIPPIC(&)FPD	2.0 pM [195]
Chymotrypsin	GRC(&)TXSIPPIC(&)FPD (X = 4-fluoro-L-phenylalanine)	30.0 pM [196]
Matriptase	KRC(&)TKSIPPRC(&)HPD (SDMI-3)	2.1 pM [197]
Plasmin	&1GRC(&2)YKSKPPIC(&2)FPD&1	50.0 pM [198]
KLK-4	&1GFC(&2)QRSIPPIC(&2)FPN&1	40.0 pM [199]
KLK-5	&1GYC(&2)NRSYPPEC(&2)FPN&1	340 pM [200]
KLK-7	&1GKC(&2)LFSNPPIC(&2)FPN&1	140 pM [201]
Furin	KRC(&)KKSIPPRC(&)F-NH2	490 pM [188]
Chymase	&1GRC(&2)QXSEPPEC(&2)FPD&1 (X = 4-chloro-L-phenylalanine)	1.8 nM [202]
Cathepsin G	&1GTC(&2)X1X2SDPPIC(&2)FPN&1 (X1 = norleucine; X2 = 4-guanidine-L-phenylalanine)	1.6 nM [203]

**Table 9:** Synthesized inhibitors and their inhibition constants against furin [189]. Residue numbering, according to Schechter and Berger [228].

No.	P5	P4	P3	P2	P1	P1′–P4′							
	1	2	3	4	5	6–9	10	11	12	13–14	C-terminus	K_i_, nM	Error, nM
**1** ^a^	G	R	C	T	K	SIPP ^c^	I	C	F	PD	OH	35,234	1579
**2** ^b^	K	R	C	T	K	SIPP	R	C	H	PD	OH	24.1	0.8
**3**	G	R	C	R	R	SIPP	R	C	H	PD	OH	1157	10
**4**	G	R	C	R	K	SIPP	R	C	H	PD	OH	29.3	0.6
**5**	K	R	C	R	K	SIPP	R	C	H	PD	OH	8.8	0.5
**6**	R	R	C	R	K	SIPP	R	C	H	PD	OH	9.1	0.3
**7**	K	R	C	R	K	SIPP	I	C	F	PD	OH	9.8	0.5
**8**	K	A	C	R	K	SIPP	R	C	H	PD	OH	1301	53
**9**	K	R	C	R	K	SIPP	R	C	H	PD	OH	2078	62
**10**	K	R	C	K	K	SIPP	R	C	H	PD	OH	3.8	0.2
**11**	K	R	C	K	K	SIPP	I	C	F		NH_2_	0.49	0.04
**12**	K	R	C	K	K	SIPP	I	C			NH_2_	0.71	0.04
**13**	K	R	C	K	K	SIPP	R	C			NH_2_	4.7	0.1
**14**	K	R	C	K	K	SIPP	A	C	F		NH_2_	3.7	0.2
**15**	K	R	A	R	K	SIPP	R	A	H	PD	OH	2373	87
**16**	K	R	Aha ^d^	K	K	SIPP	I	Pra ^e^			NH_2_	21.8	0.6
**17**	K	R	Aha	K	K	SIPP	I	Pra			NH_2_	5.0	0.4

^a^ 1 is SFTI-1. ^b^ 2 is SDMI-3. ^c^ SIPP—Ser-Ile-Pro-Pro. ^d^ Aha—L-azidohomoalanine. ^e^ Pra—l-propargylglycine.

**Table 10:** Inhibitors and their inhibition constants against furin, matriptase, and trypsin (Data taken from Table 1 and Table 2 of Fittler et al. [230]).

No.	P5	P4	P3	P2	P1	P1′–P4′				C terminal	K_i_, nM		
	1	2	3	4	5	6–9	10	11	12		Furin	Matriptase	Trypsin
**11**	K	R	C	K	K	SIPP	I	C	F	NH_2_	0.49	560	>10,000
**12**	K	R	C	K	K	SIPP	I	C		NH_2_	0.71	4528	>10,000
**16**	K	R	Aha	K	K	SIPP	I	Pra		NH_2_	21.8	9298	>10,000
**17**	K	R	Aha	K	K	SIPP	I	Pra		NH_2_	5.0	6681	>10,000

SIPP—Ser-Ile-Pro-Pro; Aha—L-azidohomoalanine; Pra—l-propargylglycine.

**Table 11:** Structures and potency of the synthesized furin inhibitors. Due to the increased length, the sequences of inhibitors **21**–**24** are given in one-letter code. Data taken from publication [233].

No.	Structure	Furin K_i_ (nM)
**2**	&[glutaryl-Arg-Arg-Lys]&-Arg-4-Amba · 4 TFA	0.504 ± 0.097
**3**	&[glutaryl-Arg-Arg-Lys]&-Lys-4-Amba · 4 TFA	1.05 ± 0.14
**4**	&[glutaryl-Arg-Arg-Arg-Lys]&-Arg-4-Amba · 4 TFA	0.68 ± 0.1
**5**	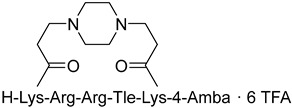	0.491 ± 0.056
**6**	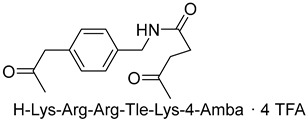	5.04 ± 1.81
**7**	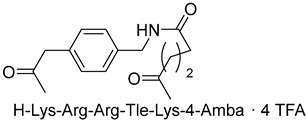	1.17 ± 0.26
**8**	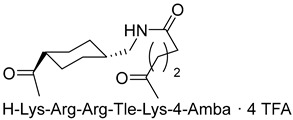	0.99 ± 0.01
**9**	&[succinyl-2-Nal-Arg-Arg-Arg-Lys]&-Arg-4-Amba · 5 TFA	0.378 ± 0.098
**10**	&[succinyl-2-Nal-Arg-Arg-Arg-Arg-Lys]&-Arg-4-Amba · 6 TFA	0.0538 ± 0.0059 ^a^
**11**	&[succinyl-2-Nal-Arg-Arg-Arg-Arg-Arg-Lys]&-Arg-4-Amba · 7 TFA	0.146 ± 0.032 ^a^
**12**	&[succinyl-Phe-2-Nal-Arg-Arg-Arg-Lys]&-Lys-4-Amba · 5 TFA	0.618 ± 0.002
**13**	&[succinyl-Phe-2-Nal-Arg-Arg-Arg-Arg-Lys]&-Lys-4-Amba · 6 TFA	0.136 ± 0.020
**14**	&[succinyl-Phe-2-Nal-Arg-Arg-Arg-Arg-Arg-Lys]&-Lys-4-Amba · 7 TFA	0.154 ± 0.047
**15**	&[Arg_6_]& · 6 TFA	110.4 ± 1.0
**16**	&[Arg_8_]& · 8 TFA	22.7 ± 0.5
**17**	&[Arg_10_]& · 10 TFA	27.8 ± 1.2
**18**	H-Arg_6_-OH · 7 TFA	9.4 ± 2.3
**19**	H-Arg_8_-OH · 9 TFA	6.0 ± 0.4
**20**	H-Arg_10_-OH · 11 TFA	9.3 ± 1.2
**21**	Ac-RQIKIWFQNRRMKWKKRVR-4-Amba · 10 TFA	19.0 (1.86 ± 0.03) ^b^
**22**	Ac-YGRKKRRQRRRVR-4-Amba · 10 TFA	11.0 (2.04 ± 0.13) ^b^
**23**	Ac-AGYLLGKINLKALAALAKKILRVR-4-Amba · 7 TFA	22.8 (1.51 ± 0.24) ^b^
**24**	Ac-RRRRRRRVR-4-Amba · 9 TFA	10.7 (1.69 ± 0.31) ^b^

^a^ K_i_ values were determined under tight-binding conditions. ^b^ The data represent IC_50_ values (average of two measurements) determined in the presence of 12.5 µM of the substrate Phac-Arg-Val-Arg-Arg-AMC; the Hill-coefficients ± SD are given in brackets.

**Table 12:** Sequences of the most active macrocyclic polypeptides synthesized and their inhibitory properties against furin.

Peptide	Sequence	IC_50_, nM	K_i_, nM
**3**	Arg-Arg-Arg-Cys(&)-Lys-Lys-Ser-Ile-Pro-Pro-Ile-Cys(&)-Phe-NH_2_	3.07 ± 0.20	0.27 ± 0.02
**5**	&^1^Lys-Arg-Arg-Cys(&^2^)-Lys-Lys-Ser-Ile-Pro-Pro-Ile-Cys(&^2^)-Phe&	2.41 ± 0.12	0.21 ± 0.01
**6**	&^1^Arg-Arg-Arg-Cys(&^2^)-Lys-Lys-Ser-Ile-Pro-Pro-Ile-Cys(&^2^)-Phe&	2.91 ± 0.17	0.25 ± 0.01
**FI**	Lys-Arg-Cys(&)-Lys-Lys-Ser-Ile-Pro-Pro-Ile-Cys(&)-Phe-NH_2_	4.42 ± 0.22	0.38 ± 0.02

**Table 13:** Guanidinylated aryl-2,5-dideoxystreptamine inhibitors of PCs [234].

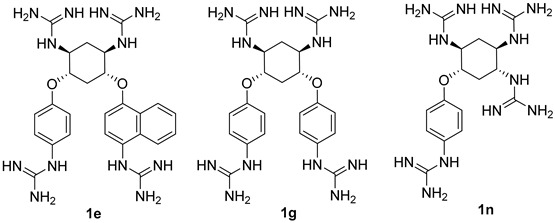							
**No.**	**Enzyme**						
	Furin	PC6B	PACE4	PC7	LF	Trypsin	MT1-MMP
	**K_i_, nM**						
**1e**	6 ± 2	4 ± 1	25 ± 2	415 ± 14	1002 ± 19	>200,000	>200,000
**1g**	12 ± 3	4 ± 0	41 ± 1	595 ± 47	1241 ± 36	>200,000	>200,000
**1n**	46 ± 3	21 ± 1	58 ± 3	1100 ± 76	3053 ± 15	>200,000	>200,000

**Table 14:** Furin inhibitors including the 2-(3,5-dichlorophenyl)-pyridinic moiety [243].

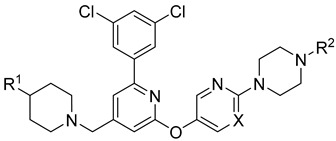						
No.	No. [243]	X	R1	R2	pIC_50_	IC_50_, nM
**1**	**18**	N	CH_2_NHCONHMe	CH(Me)CH_2_CH_2_SO_2_Me	10.7	0.020
**2**	**24**	N	CH_2_CO_2_H	1s,3s-3-OH-3-Me-cyclobutyl	10.8	0.016
**3**	**208**	N	CH_2_CO_2_NHMe	CH(Me)CH_2_CH_2_CO_2_H	10.9	0.013
**4**	**207 (BOS-318)**	N	CH_2_CO_2_H	Me	9.1	0.8
**5**	**250**	C	CH_2_CH_2_OMe	Me	8.9	1.3
**6**	**258**	C	CH_2_CH_2_CO_2_H	Me	9.6	0.3
**7**	**263**	N	CH_2_CH_2_CO_2_H	Me	9.8	0.16
**8**	**369**	N	CH(Me)CH_2_CH_2_CO_2_H	Me	9.3	0.5
**9**	**128**				7.9	13.0

**Table 15:** IC_50_ values for BOS-318 and Dec-RVKR-CMK against furin and PC subtilisin/kexin-type (PCSK) 5, 6, and 7 and in vitro Golgi inhibitory activity determined in U2OS cells [105].

PC	IC_50_, nM	
	BOS-318	Dec-RVKR-CMK
Furin	1.9 ± 1.1	1.3 ± 3.6
PCSK5	25.3 ± 4.8	0.17 ± 0.21
PCSK6	209.4 ± 62.5	0.65 ± 0.43
PCSK7	45.8 ± 25.7	0.54 ± 0.68
In vitro Golgi (U2OS), EC_50_	23.5 ± 14.7	9108 ± 6187

**Table 16:** Activity values of BOS-series furin inhibitors across various proteases.

Compounds	Furin	PC5	PACE4	PC7	Golgi (U2OS)
	IC_50_, nM				
**BOS-318**	8.8 ± 0.4	6.7 ± 0.15	6.7 ± 0.15	7.4 ± 0.22	7.7 ± 0.24
**BOS-981**	9.3 ± 0.5	7.5 ± 0.22	6.9 ± 0.1	6.9 ± 0.2	8.3 ± 0.25
**BOS-857**	9.4 ± 0.3	7.6 ± 0.2	6.7 ± 0.24	6.9 ± 0.3	7.6 ± 0.2
**Dec-RVKR-CMK**	9.1 ± 0.43	9.9 ± 0.38	9.2 ± 0.24	9.6 ± 0.63	5.1 ± 0.33

**Table 17:** Activity and toxicity of non-covalent polypeptide inhibitors of furin in mice.

No. [Ref.]	Structural Formula	K_i_, pM	In mice	
			Tolerated Dose, mg/kg	Number of Deaths at Next Higher Dose ^a^
**1 (MI-1148)** [153,158]	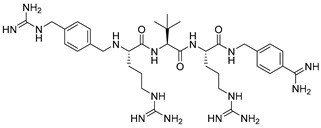	5.5	2.5	4 of 4 at 5 mg/kg
**2** [153,158]	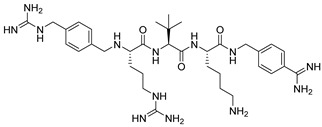	8.8	5	4 of 4 at 10 mg/kg
**5** [158]	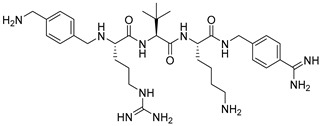	114	15	higher dose not tested
**6** [158]	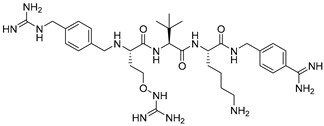	36.3	10	1 of 4 at 15 mg/kg
**8 (MI-1851)** [151,158]	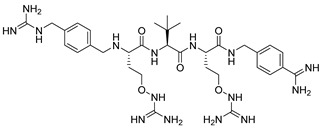	10.1	15	higher dose not tested
**17** [164]	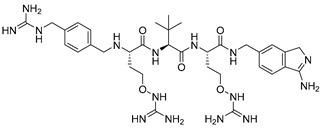	7.08	15	1 of 4 at 20 mg/kg

^a^ In each group, four mice (2 female and 2 male) were IP treated.

**Table 18:** Compound effects of furin inhibitors on mouse lung **TGFβ** and hydroxyproline content in the bleomycin-induced lung fibrosis mouse model. The % inhibition is presented at a once-daily frequency dose of 10 mg/kg drug relative to the levels induced by bleomycin in animals treated with placebo (Data taken from [95]).

No. [90]	Furin Inhibitor	pIC_50_/IC_50_	Route	% Inhibition in Lung of *	
				TGFβ	Hydroxyproline
**137**	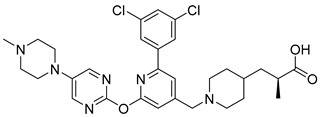	9.1/0.8 nM	Oral	81	54
**207, BOS-318**	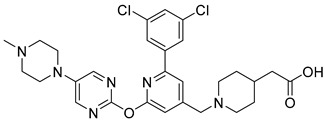	9.1/0.8 nM	Oral	66	46
**263**	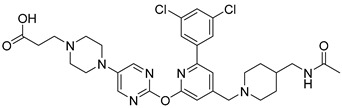	9.8/0.16 nM	IP	86	60

* *t*-test: *p* < 0.05.

## Data Availability

No new data were created or analyzed in this study. Data sharing is not applicable to this article.
